# Mediation of nitrogen by post-disturbance shelf communities experiencing organic matter enrichment

**DOI:** 10.1007/s10533-017-0370-5

**Published:** 2017-08-29

**Authors:** Marija Sciberras, Karen Tait, Guillaume Brochain, Jan G. Hiddink, Rachel Hale, Jasmin A. Godbold, Martin Solan

**Affiliations:** 1 School of Ocean Sciences, Bangor University, Askew St, Menai Bridge, Anglesey LL59 5AB UK; 2 Plymouth Marine Laboratory, Prospect Place, The Hoe, Plymouth, PL1 3DH UK; 3 Ocean and Earth Science, National Oceanography Centre Southampton, University of Southampton Waterfront Campus, European Way, Southampton, SO14 3ZH UK

**Keywords:** Ammonia-oxidisers, Bioturbation, Bottom fishing, Denitrification, Ecosystem functioning, Microbial–invertebrate interactions, Nitrogen cycling

## Abstract

Microbes and benthic macro-invertebrates interact in sediments to play a major role in the biogeochemical cycling of organic matter, but the extent to which their contributions are modified following natural and anthropogenic changes has received little attention. Here, we investigate how nitrogen transformations, ascertained from changes in archaeal and bacterial N-cycling microbes and water macronutrient concentrations ([NH_4_–N], [NO_2_–N], [NO_3_–N]), in sand and sandy mud sediments differ when macrofaunal communities that have previously experienced contrasting levels of chronic fishing disturbance are exposed to organic matter enrichment. We find that differences in macrofaunal community structure related to differences in fishing activity affect the capacity of the macrofauna to mediate microbial nitrogen cycling in sand, but not in sandy mud environments. Whilst we found no evidence for a change in ammonia oxidiser community structure, we did find an increase in archaeal and bacterial denitrifier (A*nirKa*, *nirS*) and anammox (*hzo*) transcripts in macrofaunal communities characterized by higher ratios of suspension to deposit feeders, and a lower density but higher biomass of sediment-reworking fauna. Our findings suggest that nitrogen transformation in shelf sandy sediments is dependent on the stimulation of specific nitrogen cycling pathways that are associated with differences in the composition and context-dependent expression of the functional traits that belong to the resident bioturbating macrofauna community.

**Electronic supplementary material:**

The online version of this article (doi:10.1007/s10533-017-0370-5) contains supplementary material, which is available to authorized users.

## Introduction

Marine soft-sediments cover almost 70% of the earth’s surface and play a fundamental role in the remineralization of organic carbon and nutrient cycling (Olsgard et al. 2008). According to current nitrogen budgets, it is estimated that up to 80% of the nitrogen needed by primary producers in shallow shelf seas is provided by benthic remineralization processes, primarily driven by microbial organisms that occur across the oxic/anoxic interface near the sediment surface (Dale and Prego 2002; Zehr and Kudela 2011). With 23% of the global human population concentrated in coastal areas, at an average density nearly 3 times higher than the global average (Small and Nicholls 2003) and an increase in demand in food production, coastal and shelf sea benthic systems are increasingly vulnerable to anthropogenic activities such as fishing and are at higher risk of eutrophication following excessive nutrient input. Any change in the balance and distribution of reactive nitrogen (e.g. ammonium NH_4_^+^, nitrite NO_2_^−^ and nitrate NO_3_^−^), oxygen, and organic substrates are likely to have profound consequences for nitrification, denitrification and anaerobic ammonium oxidation (anammox) that regulate coastal nitrogen budgets (Laverock et al. 2011).

Bottom fishing that uses demersal gear such as trawls and dredges to catch fish, crustaceans and bivalves living in, on or in association with the seabed, exerts a number of pressures on benthic systems that might influence sedimentary nutrient generation and budgets (Pilskaln et al. 1998; Olsgard et al. 2008). Physical processes such as sediment resuspension and sediment mixing caused by trawling alter grain size distribution, sediment sorting and porosity (Trimmer et al. 2005) that may in turn disrupt nitrification and denitrification processes (Rysgaard et al. 1994; Kitidis et al. 2017) through changes in oxygen penetration depth within the sediment (Warnken et al. 2003) and burial of organic matter to anoxic layers before aerobic remineralisation can take place (Mayer et al. 1991; Pilskaln et al. 1998). The combination of removal of surficial sediments and mixing or burial of organic matter to depth (Duplisea et al. 2001; Warnken et al. 2003) occurs on different time-scales to those of alterations in community structure; changes in pore-water and bottom water nutrient levels due to sediment resuspension return to pre-trawling levels within minutes to hours (e.g. Falcão et al. 2003; Trimmer et al. 2005; Goldberg et al. 2014), whilst microbial assemblage structure and biomass in the surficial sediment layers (upper 1 cm) return to pre-disturbance levels within days (Fiordelmondo et al. 2003) to 4–6 months (Watling et al. 2001). In contrast, macrofaunal communities can take up to 4–5 years to recover (Kaiser et al. 2006; Lambert et al. 2014). Over extended and repeated periods of fishing activity, changes in the functional composition of invertebrate communities can have a disproportionate influence on nutrient cycling through changes in the bioturbation potential of the benthic community (Duplisea et al. 2001; Widdicombe et al. 2004). The active redistribution of particles (bioturbation) and fluids (bioirrigation) by infaunal macro-invertebrates, such as bivalves, polychaetes and crustaceans, directly contributes to the spatial and temporal heterogeneity of oxic and anoxic zones (Bertics and Ziebis 2009), organic matter availability (Levin et al. 1997) and the distribution of metabolic electron acceptors (Fanjul et al. 2007) that are important in controlling microbial process rates (Gilbertson et al. 2012; Laverock et al. 2014). The close association between the macrobenthic invertebrate community composition and microbial activity suggests that a reduction in bioturbation potential of the benthic community ensuing from the loss or change in relative composition of invertebrate species associated with bottom fishing will lead to changes in microbial-mediated processes such as nitrification, denitrification and anammox. Relative to the direct physical effects of fishing gear on sediment and faunal structure, however, the extent to which microbial–invertebrate coupling is modified and affects nutrient budgets in post-disturbance communities has received little attention.

Fluctuations in organic matter input following events such as algal blooms or run-off associated with high intensity precipitation, also have a prominent influence on benthic invertebrate fauna (van Oevelen et al. 2009; Zhang et al. 2015) and microbial community composition (Franco et al. 2007; Mayor et al. 2012; Tait et al. 2015). Benthic communities may be affected positively as food becomes available to both invertebrate grazers and bacteria and archaea, or negatively because an excess of organic matter may result in deoxygenation of the sediment (Quijón et al. 2008; Mayor et al. 2012). Zhang et al. (2015) observed a doubling effect on macrofaunal production and biomass during a spring algal bloom relative to the end of the bloom in the Western English Channel. Increases in organic matter have also been related to increases in bacterial biomass (Tait et al. 2015), in bacterial growth efficiency and carbon mineralization (Mayor et al. 2012), leading to subsequent increases in NH_4_^+^ sediment flux, oxygen consumption and denitrification (Caffrey et al. 1993; Conley and Johnstone 1995). Whether post-disturbance benthic communities retain the assimilative capacity to ‘process’ system-level nutrient inputs has not been adequately evaluated.

Here, we investigate how post-disturbance macro-invertebrate communities affect archaeal and bacterial N-cycling community activity and composition and associated nutrient concentrations. We compare communities (macro-invertebrate and microbial) from sites that have been exposed to different levels of chronic fishing activity, on the a priori assumption that benthic macro-invertebrate communities would be restructured by chronic physical disturbance and that the adjusted post-disturbance community would persist long after the perturbation event (Kaiser et al. 2006). Further, we examine whether the effect of organic matter enrichment is sufficient to alter nitrogen dynamics by activating the microbial community. Our motivation was that the addition of organic matter would likely result in an increase in microbial activity, leading to increases in NH_4_^+^ sediment flux, oxygen consumption and denitrification that might be sufficient to offset any negative biogeochemical effects related to faunal change associated with prior fishing activity. To widen the generalizability of our conclusions, we examine the effects of chronic fishing disturbance and organic matter enrichment on nitrogen cycling in different sediment types; a diffusion dominated community (sandy mud) and an advection dominated community (sand). We assume that the level of biogeochemical performance that is realized in either sediment type will depend at least in part on the structure and composition of the post-disturbance macro-invertebrate community, as the active redistribution of particles and fluids by the macrofauna disproportionately influences benthic fluxes and total benthic metabolism (Mermillod-Blondin et al. 2004; Mermillod-Blondin and Rosenberg 2006).

## Methods

### Sediment collection and experimental set-up

In order to investigate how the effects of organic matter enrichment (levels: Non-enriched and Enriched) and previous exposure to bottom fishing (levels: Low and High) affect macro-invertebrate activity and microbial transformations of nitrogen in sand (S) and in sandy mud (sM) communities, we collected and maintained intact sediment cores (n = 40, 5 cores per treatment; LWH, 20 × 20 × 12 cm) with their associated fauna using a 0.1 m^2^ NIOZ (Netherlands Institute for Sea Research, Texel) corer from two fishing grounds in the Irish Sea. Sandy (S) sites were located off the east coast of the Isle of Man where scallop dredging for *Pecten maximus* and some otter trawling for *Aequipecten opercularis* occurs, whereas sand muddy (sM) sites were located off the coast of Cumbria, England, where otter trawling for *Nephrops norvegicus* and gadoid fish occurs (Table 1). Logistics such as available ship time for collecting and storage space in constant-temperature rooms for housing the mesocosms posed limitations on the number of within-treatment replicates for this study. We acknowledge that we used a relatively small number of replicates and caution that p-values close to a probability value of 0.05 should be interpreted with care; nonetheless we adopt a conservative approach and present marginal (p < 0.075) findings that indicate possible trends. Within each fishing ground, sediment cores were collected from two sites of contrasting exposure to chronic fishing disturbance (Table 1). We categorized fishing activity at each site by calculating the number of times the site is swept by bottom fishing gear in a year (km^2^ swept km^−2^ seabed year^−1^) using Vessel Monitoring System (VMS) records for UK registered vessel >15 m over the 3 year period prior to our survey (further details in Sciberras et al. 2016). Since VMS is only mandatory for vessels over 15 m (EC 2003), the activity of vessels smaller than 15 m, particularly those between 8 and 15 m is not represented. Therefore, estimates of fishing frequency may be underestimates of the actual fishing intensity, but as the spatial distribution of large and small trawlers are correlated, our measure of fishing frequency is a useful indicator of the relative fishing disturbance experienced by benthic communities at the sampled sites. Variation in habitat characteristics (e.g. sediment grain size composition, organic matter content, water depth, bottom temperature and tidal shear stress) among replicate cores collected from within each of the two sediment types was minimized to ensure that any observed differences reflected differences associated with changes in species composition due to fishing rather than environmental variability (Table 1, Electronic Supplementary Material (ESM) 1). Sediment grain size and organic matter content were determined for a separate sediment sample (ø = 5 cm, 5 cm deep) taken from each NIOZ core sample collected on-site. A combination of dry sieving (1–9.5 mm at 0.25φ intervals) and laser diffraction techniques (Malvern 2000 particle sizer, range: 0.21–1003.44 µm) were used to produce a complete particle size distribution. Organic matter content was estimated by mass loss on ignition of ~5 g of dried sediment at 550 °C for 6 h (Holme and McIntyre 1984).

**Table 1:** Summary of environmental characteristics and bottom fishing frequency of our study sites

Site code	Geographical location (latitude, longitude)	Fishing frequency (times fished per annum)	Depth (m)	Tide stress (Nm^−2^)	Wave stress (Nm^−2^)	Sand (%)	Mud (%)	Organic matter (mg)
sM-low	54.15 N, −3.63 W	3.8	26.00	0.17	0.69	33.48 ± 2.62	66.48 ± 2.62	70 ± 6.33
sM-high	54.26 N, −3.73 W	8.4	28.54	0.22	0.68	36.43 ± 1.98	63.55 ± 1.99	90 ± 12.65
S-low	54.20 N, −4.05 W	0.25	19.80	0.17	1.00	99.51 ± 0.38	0.02 ± 0.02	40 ± 6.33
S-high	54.26 N, −4.19 W	1.63	18.79	0.11	0.73	94.2 ± 0.68	4.7 ± 0.53	70 ± 9.49

Values for percent sand, percent mud and organic matter content (mg) are given as mean ± SE (*n* = 10)

Each intact sediment core was transferred to a Perspex aquarium, overlaid by ~20 cm (8 L) of ambient seawater and incubated in the laboratory in the dark at constant temperature (13 °C, approximating mean sea bottom temperature during the sampling period, 22–28th June 2015) for 1 month. The experimental period incorporated a 15 day acclimatization period prior to the addition of organic matter and a 15 day experimental period following the addition of organic matter. Enriched treatments (n = 20) received 50 mL of the microalga *Isochrysis galbana* on day 16 (concentration of ~22 cells µL^−1^; based on field observations of chlorophyll-*a* levels at ~10 m depth for the central Celtic Sea during a typical spring algal bloom, pers. comm. Dr. Alex Poulton, National Oceanography Centre, Southampton). All aquaria were aerated by bubbling with filtered air for the duration of both the acclimatization and experimental periods.

### Water nutrient, microbial and macro-invertebrate community analysis

A pre-filtered (0.45 µm, NALGENE) water sample was collected from approximately mid-point of the overlying water column of each aquarium at the end of the experiment. Absolute concentrations of ammonium [NH_4_–N], nitrite [NO_2_–N] and nitrate [NO_3_–N] were quantified using colorimetric techniques and a segmented flow nutrient autoanalyser (Bran and Luebbe, Model AAIII).

To quantify abundance and activity of N-cycling associated microbes, sediment samples (1 mL) were collected from the top 1 cm of the sediment from each core at the end of the experiment and added to a LifeGuard Soil Preservation Solution (MoBio Laboratories, Inc., Carlsbad, California, USA) and stored at −20 °C until further analysis. RNA and DNA were extracted from 0.4 g sediment samples using the RNA PowerSoil^®^ Total RNA Isolation Kit with the RNA PowerSoil^®^ DNA Elution Accessory Kit (MoBio Laboratories, Inc., Carlsbad, California, USA). Changes in the abundance of transcripts for key nitrogen cycling processes, nitrification (archaeal and bacterial ammonia monooxygenase, *amoA* that convert NH_4_^+^ into NO_2_^−^ and NO_3_^−^), denitrification (archaeal and bacterial nitrite reductase, *nirK* and *nirS* that convert NO_3_^−^ into N_2_) and anammox (hydrazine oxidoreductase, *hzo* that converts NO_2_^−^ and NH_4_^+^ into N_2_) were analysed via quantitative PCR (qPCR). In addition, as proxies for bacterial and archaeal abundance and activity, archaeal and bacterial 16S rRNA genes and 16S RNA were also quantified. Terminal Restriction Fragment Length Polymorphism (T-RFLP) was used to compare the impact of fishing frequency and organic matter addition on the composition of total and active bacterial and archaeal communities. A detailed methodology for RNA, DNA and gene extraction, qPCR and associated primers and T-RFLP is provided in ESM2.

All invertebrates were recovered (500 µm sieve), fixed and preserved in 4% formaldehyde solution for subsequent identification to the highest practicable taxonomic resolution (mostly species) and the abundance and wet weight of each taxon was measured after blotting. Tube worms were weighed excluding tubes. The values of total biomass include fragments of organisms that could not be assigned to specific taxa.

### Statistical analysis

Statistical analyses to examine the effects of fishing frequency and organic matter enrichment on benthic communities (macrofauna, microbial) and water nutrients were kept separate for sand and sandy mud, primarily because the fisheries under study at the two sediment types use different fishing gears and operate in distinct habitat types with taxonomically different communities and because the range of fishing frequency was not comparable between the two study locations (Table 1). At the sandy mud fishing ground, very low or no fishing sites were characterized by different habitat conditions (sediment composition, tide and wave stress) from sites where fishing occurred. Therefore, sampling from sites with fishing frequency comparable to that in sand for the low fishing frequency treatment would have biased our conclusions about the effects of fishing and enrichment in sandy mud. The terms ‘low’ and ‘high’ are therefore used in a relative sense.

Linear regression models (full factorial, independent nominal variables: fishing frequency F, organic matter enrichment E) were fitted for the response variables for the invertebrate community (total invertebrate density, biomass and species richness, the ratio of suspension to deposit feeders), microbial community (abundance of bacterial and archaeal nitrifiers (AOB *amoA*, AOA *amoA*), denitrifiers (A*nirKa*, *nirS*), and anammox (*hzo*)) and associated concentrations of dissolved inorganic nitrogen ([NH_4_–N], [NO_2_–N] and [NO_3_–N]). The ratio of suspension to deposit feeders was examined as an indicator of compositional and functional change, as high levels of suspension feeder mortality (relative to deposit feeders) in fished areas have been shown to reduce the benthic oxygen demand and result in higher rates of nitrification (Allen and Clarke 2007). Information on species feeding mode was obtained from the biological traits database generated from the BENTHIS project (Bolam et al. 2014, http://www.benthis.eu/en/benthis/Results.htm, accessed 16 July 2016). Further, to assess whether the sediment reworking potential of the macro-invertebrate community differed among treatments, species were classified as epifauna (E), surficial modifiers (SM), biodiffusors (B), upwards/downwards conveyors (C) and regenerator (R) following Solan et al. (2004) and updated by Queiros et al. (2013). Epifaunal organisms include species that occur predominantly above the sediment–water interface whose activities are limited to the near-surface sediment. Surficial modifiers are organisms whose activities are mostly restricted to the uppermost few centimetres of the sediment, rarely venturing above the sediment–water interface. Biodiffusors include organisms with activities that usually result in a constant and random local sediment biomixing over short distances (ca. 5 cm). Conveyors include burrow-building species that are vertically oriented in the sediment typically feeding head-down (upward conveyors) or head-up (downward conveyors) at depth in the sediment. Regenerators are excavators that dig and continuously maintain burrows in the sediment and by doing so they mechanically transfer sediment from depth to the surface (Solan et al. 2004; Kristensen et al. 2012). Density and biomass was summed to obtain the total of each reworking mode. A linear regression model incorporating the independent terms mode of sediment reworking (R_i_), fishing frequency (F), organic matter enrichment (E), and their interactions, was fitted for total density and total biomass. A significant interaction term (F:R_i_, E:R_i_ or F:E:R_i_) would indicate that changes in total density or total biomass reflect differences in response across bioturbation groups that depend on fishing frequency and/or organic matter enrichment.

Where there was evidence of violation of homogeneity of variance, the data were analyzed using a generalised least squares (GLS) estimation procedure to allow the residual spread to vary with individual independent variables (Zuur et al. 2009). To determine the optimal variance structure, we compared the full linear regression models to the equivalent GLS models incorporating specific variance structures using Akaike information criteria (AIC) and by inspection of model residual patterns. The optimal fixed-effects structure was then obtained by applying a backward selection using the likelihood ratio test obtained by maximum-likelihood (ML) estimation. Following Zuur et al. (2009), the optimal model was estimated using REML estimation. Homogeneity of residuals was established through visual examination of plotted standardized residuals versus fitted values. All analyses were performed using the *nlme* package (v. 3.1, Bates et al. 2013) in the R statistical and programming environment (R Development Core Team 2005). A summary of the linear regression models output is presented here, coefficient tables that indicate the direction and magnitude of differences among treatments are presented in supplementary material, ESM 3. Unless indicated differently, univariate results are expressed as mean ± standard error.

Differences in macrofaunal and microbial community composition associated with different fishing frequency and organic matter enrichment treatments were examined using PERMANOVA (Permutational analysis of variance). The relative contribution of species to significant effects was identified using SIMPER (Similarity percentages). All PERMANOVA and SIMPER analyses were conducted in PRIMER-E (Version 7, http://www.primer-e.com/).

## Results

### Sandy sediments

**Fig. 1:**
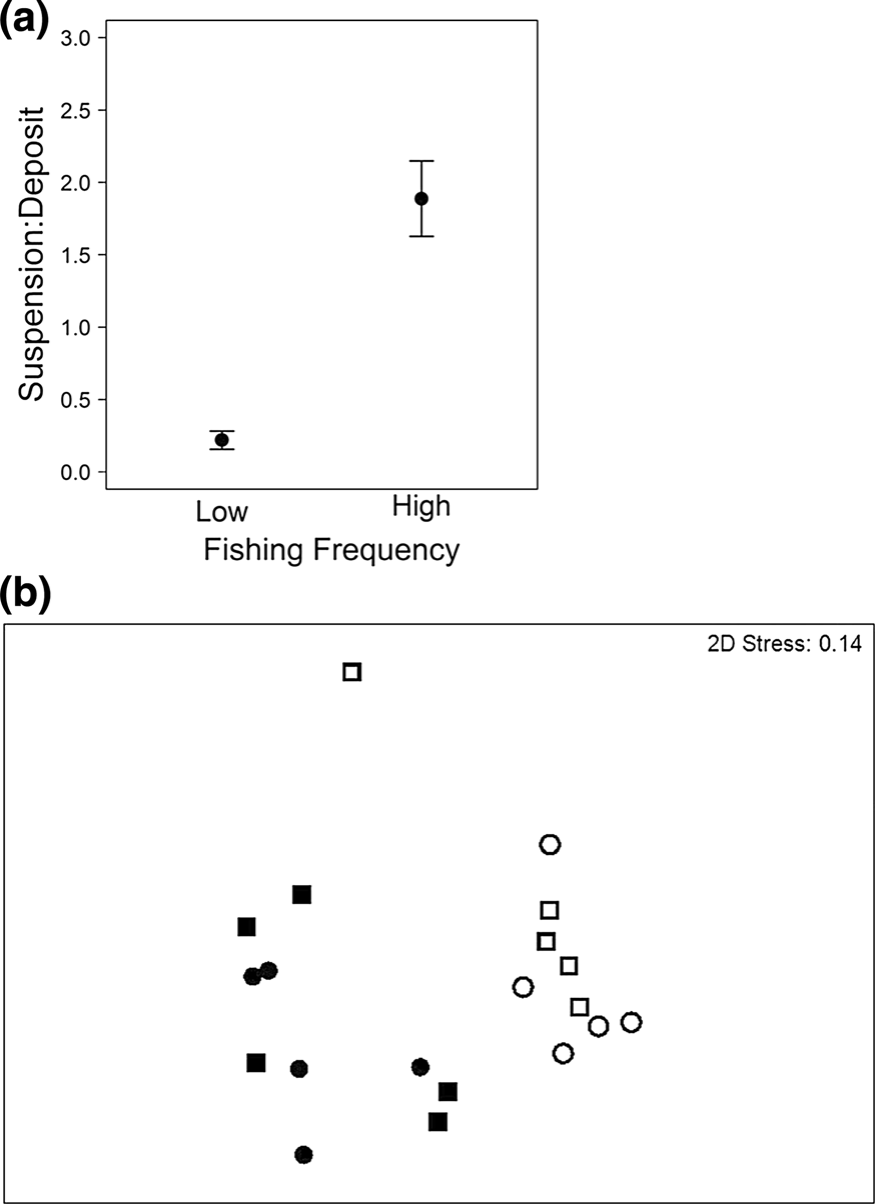
The **a** independent effect of fishing frequency on the ratio of suspension:deposit feeders (mean ± SE) and **b** a non-metric multidimensional scaling (nMDS) ordination of square-root transformed Bray–Curtis resemblance matrix of macro-invertebrate density for communities in sand. In **b** contrasting levels of bottom fishing frequency (*open symbol* low, *closed symbol* high) and organic matter enrichment (*circle* non-enriched, *square* enriched) are presented, and the MDS dimensionality representation stress value is indicated

Macro-invertebrate density ranged from 100 to 3700 ind. m^−2^, total biomass from 1 to 853.25 gWW m^−2^ and species richness from 3 to 22, but were not affected by fishing frequency or organic matter enrichment (Models 1–3, Table 2). However, the ratio of suspension to desposit feeders (Model 4, Table 2; Fig. 1a) and overall macro-invertebrate composition (PERMANOVA, density: Pseudo-F = 6.79, p = 0.001; biomass: Pseudo-F = 2.56, p = 0.009; nMDS Fig. 1b) were dependent on the frequency of fishing. The nMDS ordination for density and biomass data was very similar, therefore only that for density is presented in Fig. 1b. Deposit feeders such as the polychaete *Lagis koreni* and the echinoderms *Leptosynapta inhaerens*, *Echinocardium cordatum* and *Echinocyamus pusillus* were more abundant than suspension feeders in communities that had previously experienced a low frequency of bottom fishing (density: deposit feeders = 635 ± 155 ind. m^−2^, suspension feeders = 92.5 ± 20 ind. m^−2^), whereas suspension feeders such as *Phoronis* sp., *Owenia fusiformis* and *Abra alba* were more abundant in communities that had previously experienced a high frequency of bottom fishing (density: deposit feeders = 262.5 ± 70 ind. m^−2^, suspension feeders = 357.5 ± 47.5 ind. m^−2^) (Table 3a). Compositional differences were largely associated with a higher density of echinoderms (in particular *L. inhaerens*, *E. cordatum*, juvenile asteroids and *E. pusillus*) at lower fishing frequency, and a higher density of polychaetes (*O. fusiformis*, *Magelona* spp., *Sthenelais limicola, Ophelina acuminata* and *Chaetozone sp.*) (SIMPER, Table 3a) and larger individuals of *E. cordatum* (34 g) and the bivalves *Acanthocardia echinata* (6 g), *Chamelea striatula* (3 g) and *Thracia phaseolina* (1.4 g) at the higher fishing frequency sites (SIMPER, Table 3b).

**Table 2:** Linear regression models to examine the effects of fishing frequency and enrichment (full factorial, F × E) *in sand* (*S*), for macro-invertebrate community (Models 1–4: invertebrate density, biomass and species richness, the ratio of suspension to deposit feeders), sediment reworking groups (R_i_, reworking group density and biomass, Models 5–6), microbial community (Models 7–12: abundance of bacterial and archaeal denitrifiers (A*nirKa*, *nirS*), anammox (*hzo*), archaeal and bacterial nitrifiers (AOA *amoA*, AOB *amoA*) and ratio of bacterial and archaeal *amoA* transcripts) and associated levels of dissolved inorganic nitrogen (Models 13–15: [NO_2_–N], [NO_3_–N], [NH_4_–N])

Sediment type: SAND (S)							
Macro-invertebrate community (Initial linear model: response variable ~F × E)							
Model ID	Model	Response variable	Fishing frequency (F)	Enrichment (E)	Interaction (F:E)	Intercept only	Variance–covariate
1	GLS	Macro-invertebrate density				L = 2.54, df = 1, p = 0.11	E
2	GLS	Macro-invertebrate biomass				L = 3.37, df = 1, p = 0.07	E × F
3	GLS	Species richness				L = 1.32, df = 1, p = 0.25	E
4	GLS	suspension: deposit feeders ratio	L = 17.07, df = 1, p < 0.001				F

Sediment reworking groups (initial linear model: response variable ~F × E × Ri)									
Model ID	Model	Response variable	Fishing frequency (F)	Enrichment (E)	Reworking mode (Ri)	F:E	F:Ri	Intercept only	Variance–covariate
5	GLS	Ri density					L = 15.92, df = 4, p = 0.003		Ri × F
6	GLS	Ri biomass		L = 4.09, df = 1, p = 0.04	L = 27.05, df = 4, p < 0.0001				Ri × F

Abundance of active N-cycling associated microbes (Initial linear model: Response variable ~F × E)							
Model ID	Model	Response variable	Fishing frequency (F)	Enrichment (E)	Interaction (F:E)	Intercept only	Variance–covariate
7	GLS	*nirS*	L = 3.63, df = 1, p = 0.05	L = 11.26, df = 1, p < 0.001			F
8	GLS	*AnirKa*	L = 6.41, df = 1, p = 0.01				E × F
9	GLS	*hzo*	L = 6.59, df = 1, p = 0.01				F
10	GLS	AOA *amoA*				L = 1.25, df = 1, p = 0.26	E
11	GLS	AOB *amoA*				L = 2.50, df = 1, p = 0.11	F
12	GLS	AOB:AOA *amoA* ratio				L = 3.28, df = 1, p = 0.07	E

Water nutrient concentration (Initial linear model: Response variable ~F × E)							
Model ID	Model	Response variable	Fishing frequency (F)	Enrichment (E)	Interaction (F: E)	Intercept only	Variance–covariate
13	GLS	[NO_2_–N]	L = 5.99, df = 1, p = 0.01				F
14	GLS	[NO_3_–N]	L = 19.47, df = 1, p < 0.0001				F × E
15	GLS	[NH_4_–N]				L = 1.12, df = 1, p = 0.29	E × F

The test statistic (L-ratio or F value), degrees of freedom (df) and probability value (p) are listed for marginal (p < 0.075) or significant (p < 0.05) terms. Where all independent variables were found insignificant, we present the intercept only model. The class of variance-covariate used to specify different variances for each level of stratification within-group are also provided

**Table 3:** The similarity percentage (SIMPER) dissimilarity tables (up to 90% of cumulative differences) of taxa (a) density and (b) biomass in *sandy sites* that experienced contrasting levels of fishing frequency (Levels: low and high fishing frequency)

Species	Feeding mode	Sediment reworking functional type (Queiros et al. 2013)	Mobility (Queiros et al. 2013)	Low fishing frequency	High fishing frequency	Contr. diss. (%)
a. Groups tested: Taxon density between low and high fishing activity in sandy sediment						
*Lagis koreni*	SubDF (MarLIN 2006)	UC/DC	1	3.59	1.56	8.81
*Phoronis* sp.	PSF, ASF (MarLIN 2006)	SM	1	0.34	3.00	8.74
*Leptosynapta inhaerens*	SubDF, Det (MarLIN 2006)	SM	3	2.88	0.00	8.39
*Echinocardium cordatum*	SDF, SubDF (MarLIN 2006)	B	3	2.57	0.85	6.93
Asteroid juvenile	–	E	3	1.46	0.00	4.29
*Owenia fusiformis*	PSF, ASF, SDF, SubDF (MarLIN 2006)	SM	1	0.20	1.11	3.36
*Magelona sp.*	SDF^a^	SM	2	0.14	0.73	2.64
*Glycera oxycephala*	Pred, Scav (MarLIN 2006)	B	3	0.94	0.20	2.60
*Echinocyamus pusillus*	SDF, SubDF (MarLIN 2006)	SM	3	0.84	0.00	2.43
*Sthenelais limicola*	Pred, Scav^a^	B	3	0.10	0.71	2.28
*Ophelina acuminata*	SubDF^a^	B	3	0.20	0.76	2.27
*Chaetozone sp.*	SDF^a^	SM	2	0.00	0.64	2.07
*Magelona johnstoni*	SDF^a^	SM	2	0.20	0.66	2.03
*Dosinia lupinus*	ASF^a^, PSF	SM	2	0.54	0.00	1.94
*Poecilochaetus serpens*	SDF, SubDF, PSF, ASF (MarLIN 2006)	SM	2	0.54	0.41	1.91
*Thracia phaseolina*	PSF, ASF, SDF, SubDF (MarLIN 2006)	UC/DC	2	0.47	0.34	1.79
*Aricidea sp.*	SDF^a^, SubDF	SM	3	0.58	0.10	1.75
*Spiophanes bombyx*	PSF, ASF, SDF, SubDF (MarLIN 2006)	UC/DC	1	0.61	0.10	1.70
*Corystes crassivelanus*	Pred, Scav (MarLIN 2006)	R	4	0.40	0.60	1.67
*Venus casina*	ASF^a^, PSF	SM	2	0.44	0.30	1.62
*Ophiuroid juvenile*	–	SM	2	0.54	0.10	1.61
*Scalibregma inflatum*	SDF, SubDF (MarLIN 2006)	B	4	0.14	0.50	1.58
*Abra alba*	PSF, ASF, SDF, SubDF (MarLIN 2006)	SM	2	0.00	0.56	1.57
*Nephtys caeca*	Pred, Scav (MarLIN 2006)	B	3	0.44	0.00	1.53
*Goniada sp.*	Pred, Scav (MarLIN 2006)	B	3	0.34	0.24	1.51
*Ensis* juvenile	ASF (MarLIN 2006)	SM	2	0.34	0.20	1.22
*Sthenelais* sp.	Pred^a^, Scav	B	3	0.00	0.38	1.20
*Nephtys* sp.	Pred, Scav (MarLIN 2006)	B	3	0.10	0.34	1.15
*Spio* sp.	SDF, SubDF (MarLIN 2006)	UC/DC	2	0.30	0.20	1.14
Terebellidae	–	UC/DC	1	0.20	0.24	1.09
Syllidae	–	B	3	0.00	0.30	1.05
*Abra prismatica*	PSF, ASF, SDF, SubDF (MarLIN 2006)	SM	2	0.24	0.00	0.79
*Gattyana cirrhosa*	Pred, Scav (MarLIN 2006)	B	3	0.10	0.20	0.76
Nematoda	–	SM	2	0.00	0.24	0.75
*Scolelepis squamata*	SDF^a^	UC/DC	2	0.24	0.00	0.70
*Scoloplos armiger*	SubDF^a^	B	3	0.24	0.00	0.70
Sabellidae	–	SM	1	0.10	0.20	0.64
*Bathyporeia gracilis*	SDF^a^, SubDF	SM	3	0.20	0.00	0.57
*Orbinia* sp.	SubDF^a^	B	3	0.10	0.10	0.57
*Pagurus* sp.	SDF, Pred, ASF (MarLIN 2006)	E	4	0.00	0.20	0.53
*Cerebratulus* sp.	Pred^a^, Scav	B	3	0.20	0.00	0.52
b. Groups tested: Taxon biomass between low and high fishing activity in sandy sediment						
*Echinocardium cordatum*	SDF, SubDF (MarLIN 2006)	B	3	0.28	1.06	19.76
*Corystes crassivelanus*	Pred, Scav (MarLIN 2006)	R	4	0.30	0.29	11.17
*Thracia phaseolina*	PSF, ASF, SDF, SubDF (MarLIN 2006)	UC/DC	2	0.05	0.25	6.19
*Lagis koreni*	SubDF(MarLIN 2006)	UC/DC	1	0.23	0.12	6.06
*Acanthocardia echinata*	PSF, ASF (MarLIN 2006)	SM	2	0.00	0.24	4.94
*Chamelea striatula*	PSF, ASF (MarLIN 2006)	SM	2	0.00	0.17	4.41
*Nephtys incisa*	SDF, SubDF (MarLIN 2006)	B	3	0.09	0.03	3.06
*Glycera oxycephala*	Pred, Scav (MarLIN 2006)	B	3	0.11	0.01	2.94
*Phoronis sp.*	PSF, ASF (MarLIN 2006)	SM	1	0.01	0.1	2.56
*Sigalion mathilde*	Pred^a^, Scav	B	3	0.00	0.06	2.13
*Lumbrineris sp.*	Pred, Scav (MarLIN 2006)	B	3	0.00	0.07	1.96
*Ophelina acuminata*	SubDF^a^	B	3	0.01	0.06	1.92
*Owenia fusiformis*	PSF, ASF, SDF, SubDF (MarLIN 2006)	SM	1	0.01	0.07	1.91
*Sthenelais limicola*	Pred^a^, Scav	B	3	0.01	0.05	1.88
*Venus casina*	ASF^a^, PSF	SM	2	0.03	0.07	1.85
*Scolelepis squamata*	SDF^a^	UC/DC	2	0.06	0.00	1.38
*Pagurus sp.*	SDF, Pred, ASF (MarLIN 2006)	E	4	0.00	0.04	1.36
*Abra alba*	PSF, ASF, SDF, SubDF (MarLIN 2006)	SM	2	0.00	0.07	1.26
*Abra prismatica*	PSF, ASF, SDF, SubDF (MarLIN 2006)	SM	2	0.04	0.00	1.26
Asteroid juvenile	–	E	3	0.04	0.00	1.22
*Cerianthus* sp.	PSF, Pred (MarLIN 2006)	SM	1	0.00	0.05	1.18
*Nucula hanleyi*	SubDF, Det (MarLIN 2006)	SM	3	0.00	0.05	1.01
*Dosinia lupinus*	ASF^a^, PSF	SM	2	0.03	0.00	0.95
*Glycera alba*	Pred, Scav (MarLIN 2006)	B	3	0.02	0.01	0.88
*Travisia forbesi*	SDF, SubDF (MarLIN 2006)	B	3	0.03	0.00	0.87
*Ensis* juvenile	ASF (MarLIN 2006)	SM	2	0.00	0.03	0.82
*Scalibregma inflatum*	SDF, SubDF (MarLIN 2006)	B	4	0.01	0.02	0.80
*Gattyana cirrhosa*	Pred, Scav (MarLIN 2006)	B	3	0.02	0.02	0.77
*Sthenelais* sp.	Pred^a^, Scav	B	3	0.00	0.05	0.77

Information on species feeding mode/s (SDF for surface deposit feeder; SubDF for subsurface deposit feeder; ASF for active suspension feeder; PSF for passive suspension feeder; Pred for predator; Scav for scavenger; Det for detritivore; feeding mode was not allocated to taxon level higher than genus and are denoted by “–”), sediment reworking functional type (E for epifauna; SM for surficial modifiers; UC/DC for upward and downward conveyors; B for biodiffusors; and R for regenerators) and mobility (*1* for organisms that live in fixed tubes; *2* indicates limited movement; *3* indicates slow, free movement through the sediment matrix; *4* indicates free movement via burrow system) are provided

^a^Biological traits database developed under the BENTHIS (Benthic Ecosystem Fisheries Impact Studies) project. [16/07/2016]. http://www.benthis.eu/en/benthis/Results.htm

**Fig. 2:**
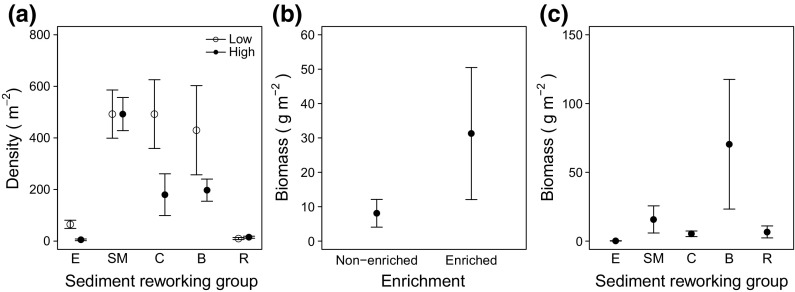
The **a** interactive effects of sediment reworking group identity and the frequency of fishing on sediment reworking group density, and **b**, **c** the independent effects of organic matter enrichment and sediment reworking group identity on sediment reworking group biomass in sandy sediments (mean ± SE). In **a** contrasting levels of bottom fishing frequency (*open symbol* low, *closed symbol* high) are presented. In **a** and **c** sediment reworking groups include epifauna (*E*), surficial modifiers (*SM*), conveyors (*C*), biodiffusors (*B*) and regenerators (*R*)

Sediment reworking group density depended on the interactive effects of sediment reworking group identity and the frequency of fishing (Model 5, Table 2); biodiffusor (e.g. *E. cordatum*, *Glycera oxycephala*) and conveyor species (e.g. *L. koreni*, *T. phaseolina*, *Spiophanes bombyx*) were more abundant after a low frequency of fishing (density: 492.5 ± 133.28 and 430 ± 172.92 ind. m^−2^, respectively) than they were after a high frequency of fishing (density: 180 ± 80.78 and 197.5 ± 42.89 ind. m^−2^, respectively) (Fig. 2a). Sediment reworking group biomass, however, was dependent on the independent effects (Model 6, Table 2) of organic matter enrichment (Fig. 2b) and sediment reworking group identity (Fig. 2c), with a greater biomass attributed to biodiffusors (e.g. *E. cordatum, Sigalion mathilde, Lumbrineris* sp.) and when sediments were enriched with organic matter.

**Fig. 3:**
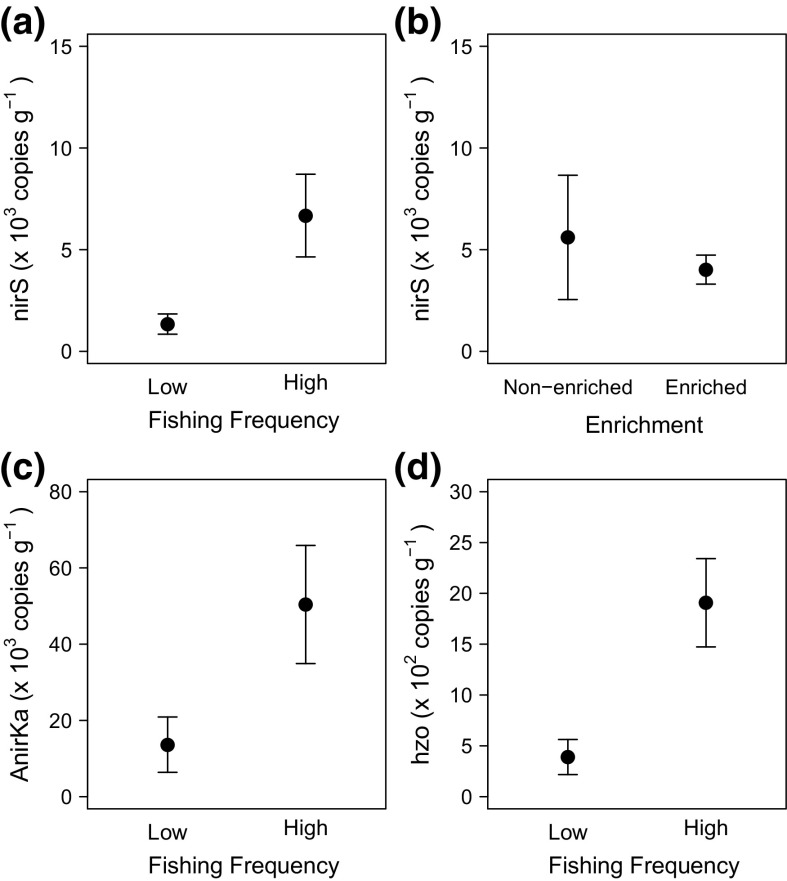
The independent effects of **a** fishing frequency and **b** organic matter enrichment on abundance of the metabolically active bacterial denitrifier (*nirS*) and the independent effect of fishing frequency on **c** archaeal (A*nirKa*) denitrifiers and **d** anammox (*hzo*) hydrazine oxidoreductase transcripts in sandy sediments. Values plotted are mean ± SE

T-RFLP profiling for archaeal and bacterial 16S rRNA genes revealed significant differences in the total microbial community structure between communities that experienced low and high frequency of fishing activity (PERMANOVA: Archaea, Pseudo-F = 2.22, p = 0.05; Bacteria, Pseudo-F = 4.03, p = 0.03), but not among enriched and non-enriched treatments (Archaea, Pseudo-F = 0.66, p = 0.61; Bacteria, Pseudo-F = 0.92, p = 0.42; ESM4 Fig. S2). The density of the metabolically active bacterial denitrifier (*nirS*) was dependent on the independent effects of fishing frequency (Fig. 3a) and organic matter enrichment (Fig. 3b), whilst both archaeal (A*nirKa*) denitrifiers (Fig. 3c) and anammox (*hzo*) hydrazine oxidoreductase transcripts (Fig. 3d) were dependent solely on the effect of fishing frequency (Models 7–9, Table 2). We found no evidence that the density of bacterial (AOB *amoA*) or archaeal (AOA *amoA*) ammonia oxidisers, or ammonia oxidiser community structure (AOB:AOA *amoA* ratio) were affected by the frequency of fishing or the level of organic matter enrichment (Models 10–12, Table 2).

**Fig. 4:**
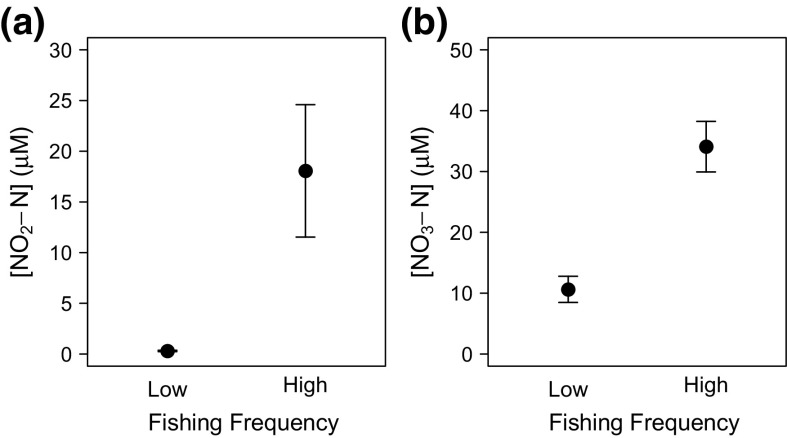
The independent effect of fishing frequency on **a** [NO_2_–N] and **b** [NO_3_–N] in sandy sediments. Values plotted are mean ± SE

For nutrients, we found that [NO_2_–N] and [NO_3_–N] were dependent on an independent effect of fishing frequency (Models 13–14, Table 2; Fig. 4), whilst [NH_4_–N] was not affected by fishing frequency or organic matter enrichment (Model 15, Table 2).

### Sandy mud sediments

**Fig. 5:**
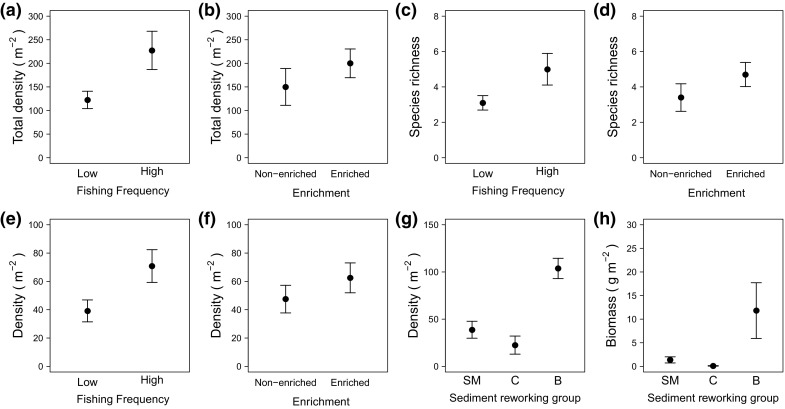
The independent effects of fishing frequency and organic matter enrichment on **a**, **b** total macrofaunal density **c**, **d** species richness and **e**, **f** sediment reworking group density. Sediment reworking group density **g** and biomass **h** were dependent on sediment reworking group identity (*SM* surficial modifiers, *C* conveyors, *B* biodiffusors). There were no species for sediment reworking groups E (epifauna) and R (regenerators) in sandy mud. Values plotted are mean ± SE

**Fig. 6:**
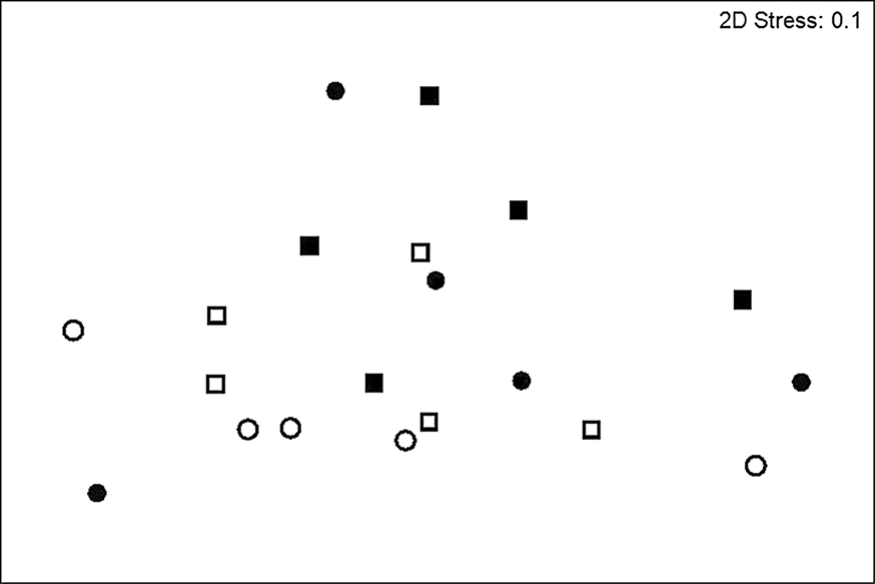
Non-metric multidimensional scaling (nMDS) ordination of square-root transformed Bray–Curtis resemblance matrix of macro-invertebrate density for communities in sandy mud that experienced contrasting levels of bottom fishing frequency (*open symbol* low, *closed symbol* high) and of organic enrichment (*circle* non-enriched, *square* enriched). MDS dimensionality representation stress value = 0.1

Relative to sandy sediments, total density (25–450 ind. m^−2^), total biomass (0.8–119.8 gWW m^−2^) and species richness (1–9) were lower in sandy mud macro-invertebrate communities. Total macro-invertebrate density (Model 16, Table 4; Fig. 5a, b) and species richness (Model 17, Table 4; Fig. 5c, d) were dependent on the independent effects of fishing frequency and organic matter enrichment. Mean total density and species richness were highest for communities that had experienced a greater frequency of fishing (density: 227.5 ± 40.5 m^−2^; species richness: 5.00 ± 0.89 core^−1^) or organic matter enrichment (density: 200.00 ± 30.51 m^−2^, species richness: 4.70 ± 0.68 core^−1^). In contrast, we found no evidence of any macro-faunal response in terms of biomass (Model 18, Table 4). Similarly, the ratio of suspension to deposit feeders did not differ between any of our treatments (Model 19, Table 4). Sediment reworking group density (Model 20, Table 4), however, reflected the independent effects of fishing frequency (Fig. 5e), organic matter enrichment (Fig. 5f) and sediment reworking group identity (Fig. 5g), whilst sediment reworking group biomass was influenced solely by sediment reworking group identity (Model 21, Table 4 with a greater biomass attributed to biodiffusors (e.g. *Goneplax rhomboides*, *Nepthys* sp.) (Fig. 5h). Nevertheless, community composition did not differ between sites of low and high fishing frequency (PERMANOVA, density: Pseudo-F = 1.30, p = 0.25; biomass, Pseudo-F = 1.37, p = 0.22, ESM 5) or between enriched and non-enriched treatments (density: Pseudo-F = 0.82, p = 0.55; biomass: Pseudo-F = 0.74, p = 0.63) (Fig. 6).

**Table 4:** Linear regression models to examine the effects of fishing frequency and enrichment (full factorial, F × E) *in sandy mud* (*sM*), for macro-invertebrate community (Models 16–19: invertebrate density, biomass and species richness, the ratio of suspension to deposit feeders), sediment reworking groups (R_i_, reworking group density and biomass, Models 20–21), microbial community (Models 22–27: abundance of bacterial and archaeal denitrifiers (*nirS*, A*nirKa*), anammox (*hzo*), archaeal and bacterial nitrifiers (AOA *amoA*, AOB *amoA*) and ratio of bacterial and archaeal *amoA* transcripts) and associated levels of dissolved inorganic nitrogen (Models 28–30: [NO_2_–N], [NO_3_–N], [NH_4_–N])

Sediment type: sandy Mud (sM)							
Macro-invertebrate community (Initial linear model: response variable ~F × E)							
Model ID	Model	Response variable	Fishing frequency (F)	Enrichment (E)	Interaction (F: E)	Intercept only	Variance–covariate
16	GLS	Macro-invertebrate density	L = 5.27, df = 1, p = 0.02	L = 4.31, df = 1, p = 0.04			F
17	GLS	Species richness	L = 3.83, df = 1, p = 0.05	L = 7.75, df = 1, p = 0.005			F
18	GLS	Macro-invertebrate biomass				L = 1.29, df = 1, p = 0.26	F × E
19	LM	suspension: deposit feeders ratio				F = 12.33, df = 16, p = 0.77	–

Sediment reworking groups (Initial linear model: Response variable ~F × E × Ri)									
Model ID	Model	Response variable	Fishing frequency (F)	Enrichment (E)	Reworking mode (Ri)	F:E	F:Ri	Intercept only	Variance–covariate
20	GLS	Ri density	L = 8.51, df = 1, p = 0.004	L = 5.59, df = 1, p = 0.02	L = 54.17, df = 1, p < 0.0001				F × E
21	GLS	Ri biomass			L = 8.23, df = 1, p = 0.02				F × Ri

Abundance of active N-cycling associated microbes (Initial linear model: response variable ~F × E)							
Model ID	Model	Response variable	Fishing frequency (F)	Enrichment (E)	Interaction (F:E)	Intercept only	Variance–covariate
22	LM	*nirS*				F = 0.005, df = 17, p = 0.94	**–**
23	LM	*AnirKa*	F = 3.86, df = 1, p = 0.07	F = 3.97, df = 1, p = 0.06			–
24	LM	*hzo*				F = 0.33, df = 17, p = 0.57	–
25	LM	AOA *amoA*				F = 0.46 df = 17, p = 0.51	–
26	LM	AOB *amoA*				F = 0.17, df = 17, p = 0.68	–
27	LM	AOB: AOA *amoA* ratio		F = 13.33, df = 1, p = 0.002			–

Water nutrient concentration (Initial linear model: response variable ~F × E)							
Model ID	Model	Response variable	Fishing frequency (F)	Enrichment (E)	Interaction (F:E)	Intercept only	Variance–covariate
28	GLS	[NO_2_–N]				L = 1.37, df = 1, p = 0.24	F × E
29	LM	[NO_3_–N]				F = 0.49, df = 19, p 0.49	–
30	GLS	[NH_4_–N]				L = 1.73, df = 1, p = 0.19	F × E

The test statistic (L-ratio or F value), degrees of freedom (df) and probability value (p) are listed for marginal (p < 0.075) or significant (p < 0.05) terms. Where all independent variables were found insignificant, we present the intercept only model. The class of variance-covariate used to specify different variances for each level of stratification within-group are also provided

**Fig. 7:**
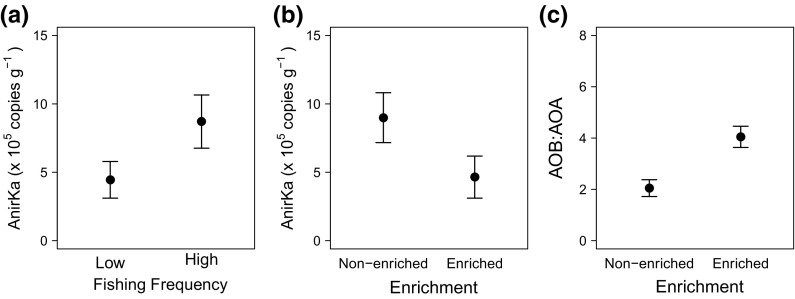
The independent effects of **a** fishing frequency and **b** organic matter enrichment on archaeal (*AnirKa*) denitrifiers, and of **c** organic matter enrichment on the ratio of bacterial to archaeal ammonia oxidisers (AOB:AOA *amoA*). Values plotted are mean ± SE

T-RFLP profiling for archaeal and bacterial 16S rRNA genes did not reveal any differences in total microbial community structure that related to either fishing frequency (PERMANOVA, Archaea: Pseudo-F = 1.07, p = 0.37; Bacteria: Pseudo-F = 1.47, p = 0.15), or organic matter enrichment (Archaea: Pseudo-F = 0.23, p = 0.95; Bacteria: Pseudo-F = 1.29, p = 0.24) (ESM4 Fig. S3). Indeed, we were unable to find any evidence supporting the view that bacterial denitrifers (*nirS* transcripts, range: 903–6330 copies g^−1^ sediment), anammox (*hzo* transcripts, range: 3160–165,000 copies g^−1^ sediment) or bacterial (AOB *amoA* transcripts, range: 1270–251,000 copies g^−1^ sediment) or archaeal ammonia oxidisers (AOA *amoA* transcripts, range: 2700–83,900 copies g^−1^ sediment) respond to differences in fishing frequency or organic matter enrichment (Models 22, 24–26, Table 4). In contrast, however, archaeal (A*nirKa*) denitrifiers did respond (Model 23, Table 4) positively to the effects of increasing fishing frequency (Fig. 7a) and negatively to increasing organic matter enrichment (Fig. 7b), although these effects were independent of one another. We also found evidence that the mean ratio of bacterial to archaeal ammonia oxidisers (AOB: AOA *amoA*) increased with organic matter enrichment (Model 27, Table 4, Fig. 7c).

Despite changes in the microbial and macrofaunal attributes of our sandy mud communities, [NH_4_–N], [NO_2_–N] and [NO_3_–N] were not affected by fishing frequency or organic matter enrichment (Models 28–30, Table 4).

## Discussion

Our findings suggest that nitrogen transformation in shelf sea sediments is dependent on whether specific microbial transcripts are influenced by differences in the composition of the bioturbating macrofauna, environmental context (here, nutrient enrichment and sediment type), and recent history of anthropogenic disturbance (here, frequency of bottom fishing), although these effects are not necessarily interactive and their relative importance is context dependent (Wohlgemuth et al. 2017). We find that the modification of invertebrate community structure following bottom fishing is particularly important for the mediation of biogeochemical processes and is not necessarily offset by the effects of organic matter enrichment on microbial composition and activity. These effects were observed in sand but not in sandy mud (where the range of fishing frequency from bottom-towed fishing gear was higher than that in the sandy area). In sand, we found that sediment characterized by higher ratios of suspension to deposit feeders and a lower density but higher biomass of bioturbating fauna, was associated with increased activity of denitrifying archaea and bacteria (A*nirKa*, *nirS*) and anammox (*hzo*) and higher levels of bottom water [NO_2_–N] and [NO_3_–N]. The higher biomass of bioturbating species, in particular *Echinocardium cordatum*, *Acanthocardia echinata* and *Chamelea striatula* and the higher density of bioirrigating tube-building species such as *Phoronis* sp. and *Owenia fusiformis,* offers an explanation for the enhanced denitrifier activity. *E. cordatum* is known to displace large volumes of sediment (20,000 cm^3^ m^−2^ day^−1^ by 40 individuals m^−2^, Lohrer et al. 2005) and although the shallow-burying bivalves *A. echinata* and *C. striatula* and the tube-building polychaetes do not build extensive burrow systems deep within the sediment, their active mixing of the uppermost sediment layers and their dominating biomass, means that their bioturbation activities are likely to have stimulated microbial denitrification and anammox, possibly by increasing the flux of [NO_2_–N] and [NO_3_–N] across the water–sediment interface, which constitutes the substrate for nitrite reductase and anammox (Howe et al. 2004; Dang et al. 2010). Measured sediment particle reworking rates using fluorescent sediment profile imaging techniques at the same study sites confirm more intense particle reworking activity in communities that have previously experienced a higher frequency of bottom fishing, substantially extending the maximum depth of sediment reworking (low frequency fishing, 1.99 ± 0.19 cm; high frequency fishing, 4.64 ± 0.5 cm) and increasing the volume of sediment available for nitrification (Hale et al. 2017). It is interesting to note that, although some bioturbation groups—such as biodiffusors and upwards/downwards conveyors—were, on average, twice as abundant at sites with a history of low frequency of fishing, the biomass of biodiffusors and surficial modifiers was substantively higher (24 times and 60 times higher, respectively) at sites with a history of high frequency of fishing. It appears that biomass had an overriding effect over density; larger individuals with a greater *per capita* effect on sediment mixing have a disproportionate effect on microbial activity and composition and, in turn, nutrient concentrations (Osinga et al. 1995; Bird et al. 1999). The increased abundance of active suspension feeders (relative to deposit feeders) is also likely to have stimulated microbial denitrification and anammox, through an increase in the provision of [NO_2_–N] and [NO_3_–N] as water is actively moved into the sediment during feeding (Howe et al. 2004; Dang et al. 2010). Although we may conclude that macrofaunal bioturbation and bioirrigation activities had a stimulatory effect on microbial denitrification, it remains unclear why we did not detect any change in archaeal or bacterial nitrifier (*amoA* gene) abundance in either sand or sandy mud sediments.

Given the findings elsewhere that report higher mortality of suspension feeders at locations that are subject to fishing activity (e.g. Tillin et al. 2006; van Denderen et al. 2015), it is surprising that we found a higher ratio of suspension to deposit feeders in sand communities that experienced a higher frequency of chronic fishing. However, a clear trend that emerges from previous studies is that the degree of natural disturbance in which a community develops determines the degree to which it is affected by bottom fishing (Kaiser and Spencer 1996; Hiddink et al. 2006; Sciberras et al. 2013). The macro-invertebrate communities at our sandy study sites are adapted to living in physically dynamic areas that are characterized by relatively high near-bed current flows (Hiddink et al. 2009) and infrequent fishing activity (1.63 times year^−1^), so community recruitment and growth is unlikely to be significantly affected by fishing. In contrast, the macro-invertebrate communities of our sandy mud sites show substantive compositional changes that relate to a fishing frequency of 3.8 times year^−1^, such that further increases in fishing activity (8.4 times year^−1^) have proportionally less effect on microbial and/or macrofaunal community composition and structure. We recognize that our study would benefit from additional locations where bottom fishing is absent, but such areas were not comparable as they were characterized by very different habitat conditions (sediment composition, tide and wave stress). In agreement with Braeckman et al. (2014), however, we find that that benthic functional diversity (expressed as community bioturbation potential, BP_C_) had a strong influence on biogeochemical cycling (sediment community oxygen consumption, denitrification rates, alkalinity and NH_4_ fluxes) in sandy sediments with high BP_C_ but not in muddy sediments, where the BP_C_ was found to be significantly lower than in sand.

We hypothesized that the addition of organic matter would increase microbial activity (measured here as the gene transcript abundance). Thus, the correlation between enrichment and macrofaunal density and species richness in sandy mud and the relative biomass of different functional groups in sand was unexpected (given the short time scale of the experiment) and most likely the result of stochastic variation in the abundance of infauna and unrelated to the enrichment treatment. However, in sandy sediments, organic matter enrichment correlated with a reduction in the variation and mean activity of bacterial denitrifiers (*nirS*), and in sandy mud sediments with a reduction in mean activity of archaeal denitrifiers (A*nirKa*), and a change in ammonia oxidiser community structure, altering the ratio of ammonia oxidising bacteria to ammonia oxidising archaea (as in Gilbertson et al. 2012). An effect, however, was not found for all microbes measured, for example for the nitrifiers (AOB *amoA*, AOA *amoA*) and anammox (*hzo*) in either sand or sandy mud. A number of studies have reported increases in bacterial biomass and activity upon addition of organic material within days or even hours (e.g. Luna et al. 2002; Gihring et al. 2009). However, others have reported a delayed microbial response (~1–2 weeks) (Tait et al. 2015) as instead of feeding directly on sinking phytodetrital material, benthic microbes may consume the organic matter released via the grazing activity of deposit and suspension feeders, thus explaining the lack of response for some microbes in our study.

The lesser importance of organic matter enrichment relative to fishing frequency related changes in macrofaunal composition documented here, may be emphasising habitat-specific differences in organic matter incorporation rate and/or differences in the response time of different components of the benthic community. Our study highlights the importance of understanding the response of multiple ecosystem components over the longer term if we are to provide ecosystem-relevant evidence to underpin decisions that aim to secure the protection of natural capital (Pittman and Armitage, 2016), and ensure the sustainable management of coastal and shelf sea ecosystem services (Voss et al. 2013).

## Electronic supplementary material

Below is the link to the electronic supplementary material.

Supplementary material 1 (DOCX 768 kb)
