# Supplementary material for: Mediation of nitrogen by post-disturbance shelf communities experiencing organic matter enrichment
Source: Biogeochemistry. 2017 Aug 29;135(1):135–53. doi: 10.1007/s10533-017-0370-5 (PMC6961516; doi:10.1007/s10533-017-0370-5)
Supplement: Supplementary file 1 — Supplementary material 1 (DOCX 768 kb) [file 10533_2017_370_MOESM1_ESM.docx]

**Running head:** Community mediation of sediment nitrogen cycling

**Article type:** General research

**Title:** Mediation of nitrogen by post-disturbance shelf communities experiencing organic matter enrichment

**Authors:** Marija Sciberras^1^, Karen Tait^2^, Guillaume Brochain^2^, Jan G. Hiddink1, Rachel Hale^3^, Jasmin A. Godbold^3^, Martin Solan^3^

**Affiliations:**

^1^ School of Ocean Sciences, Bangor University, Askew St, Menai Bridge, Anglesey, LL59 5AB, UK

^2^ Plymouth Marine Laboratory, Prospect Place, The Hoe, Plymouth, PL1 3DH, UK

3 Ocean and Earth Science, National Oceanography Centre Southampton, University of Southampton Waterfront Campus, European Way, Southampton, SO14 3ZH, UK

**Correspondence address:**

Marija Sciberras

School of Ocean Sciences, Bangor University, Askew St, Menai Bridge, Anglesey, LL59 5AB, UK

T: (0044) 01248388150

Email: m.sciberras@bangor.ac.uk

**Electronic supplementary material (ESM)**

**ESM1. Sediment characteristics of different treatment cores**

A 2-way interaction PERMANOVA model (Fishing frequency × Organic enrichment) was carried out on normalized sediment characteristic data (% gravel, % sand, % mud and organic matter content (grams)) to examine whether sediment characteristics differed significantly between treatments, as this may otherwise confound the effect of fishing frequency and organic enrichment on macrofauna, microbes and water nutrients.

None of the main terms and interaction term was significant for either sandy Mud (Table S1a) or sand (Table S1b), indicating that there are no significant differences in sediment composition among any of the fishing frequency and organic enrichment treatments. The percentage composition of gravel, sand and mud and the organic matter content (g) for each treatment combination is shown in Figure S1.

**Supplementary Table S1**. Output of the interactive PERMANOVA model (F × E) examining for differences in the sediment composition (in terms of % gravel, % sand, % mud, organic matter content) between low and high fishing frequency and enriched and non-enriched treatments in (a) sandy mud and (b) sand. df – degrees of freedom; SS – Sum of Squares; MS - Mean squares; Pseudo-F - F-value by permutation and P(perm) – probability of statistical significance based on 9,999 permutations of the data are given.

| **(a) sandy Mud (sM) cores** | | | | | |
| --- | --- | --- | --- | --- | --- |
| **Source** | **df** | **SS** | **MS** | **Pseudo-F** | **P (perm)** |
| Fishing intensity (FI) | 1 | 2.34 | 2.34 | 2.09 | 0.14 |
| Enrichment (E) | 1 | 0.30 | 0.29 | 0.27 | 0.69 |
| FI × E | 1 | 0.49 | 0.49 | 0.44 | 0.58 |
| Res | 16 | 17.85 | 1.12 |  |  |
| Total | 19 | 20.98 |  |  |  |
| **(b) sandy (S) cores** | | | | | |
| **Source** | **df** | **SS** | **MS** | **Pseudo-F** | **P (perm)** |
| Fishing intensity (FI) | 1 | 5.31 | 5.31 | 2.26 | 0.15 |
| Enrichment (E) | 1 | 2.98 | 2.99 | 1.28 | 0.31 |
| FI × E | 1 | 0.01 | 0.01 | 0.01 | 0.90 |
| Res | 16 | 37.58 | 2.35 |  |  |
| Total | 19 | 45.88 |  |  |  |

**Supplementary Figure S1.** Percentage composition of (a, e) gravel, (b, f) sand, (c, g) mud and (d, h) organic matter content (grams) at low and high fishing frequency in organically non-enriched treatments (L-NonEn, H-NonEn, respectively) and low and high fishing frequency in organically enriched treatments (L-En, H-En, respectively) in sandy Mud (a, b, c, d) and sand (e, f, g, h). Each boxplot indicates the median and interquartile range, the whiskers indicate the upper and lower quartile of data, dots represent individual observations.


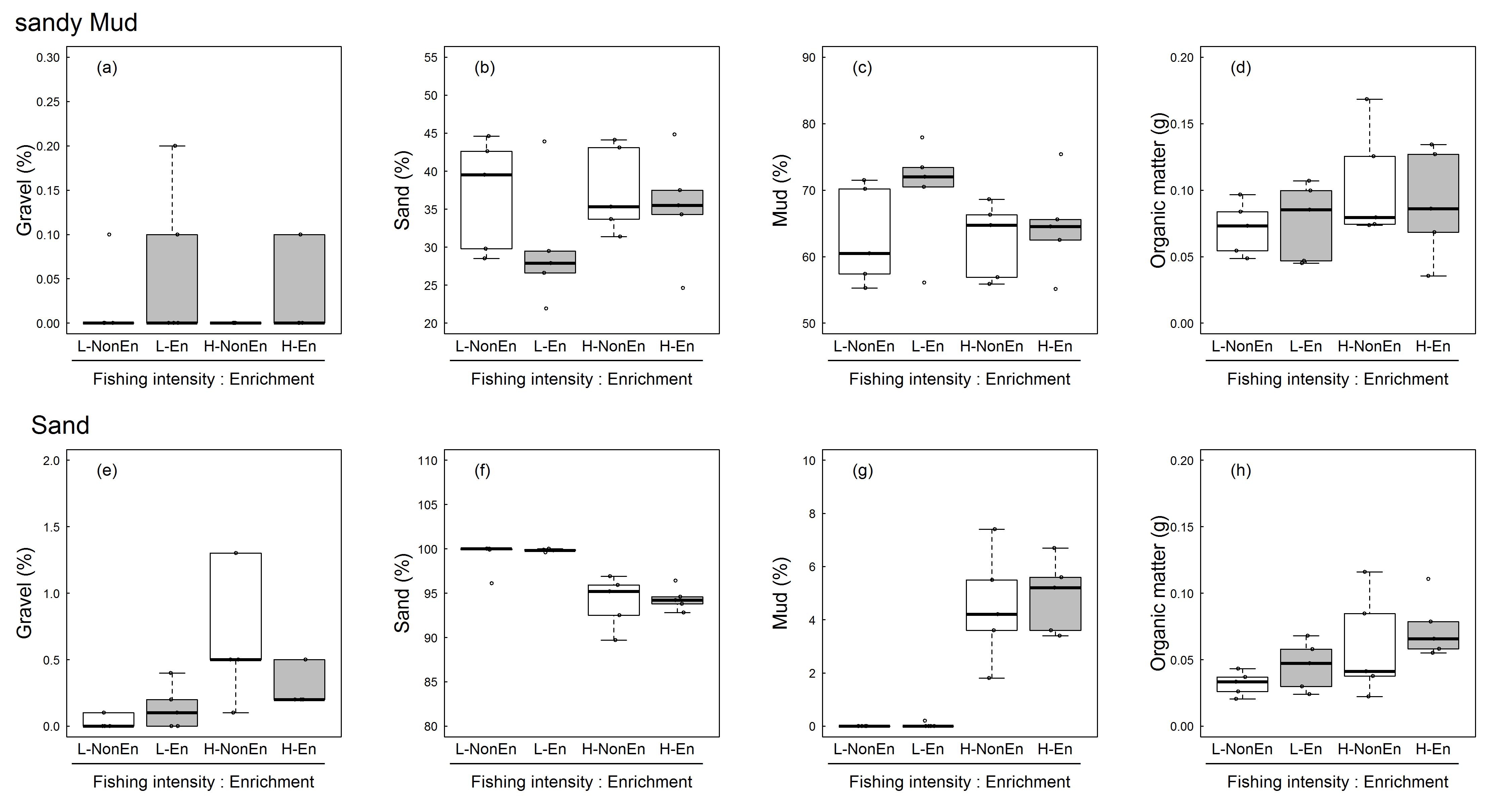


**ESM2. Detailed methodology for microbial analyses: extraction, qPRC, T-RFLP**

To quantify abundance and activity of N-cycling associated microbes, sediment samples (1 mL) were collected from the top 1 cm of the sediment from each core and added to a LifeGuard Soil Preservation Solution (MoBio Laboratories, Inc., Carlsbad, California, USA) and stored at -20˚C until further analysis. RNA and DNA were extracted from 0.4g sediment samples using the RNA PowerSoil® Total RNA Isolation Kit with the RNA PowerSoil® DNA Elution Accessory Kit (MoBio Laboratories, Inc., Carlsbad, California, USA) following the instructions of the manufacturer, and eluted in 200µL Rnase- and Dnase-free water. RNA was subsequently stored at -80˚C and DNA at -20˚C. RNA samples were reverse transcribed using the QuantiTect® Reverse Transcription (RT) Kit (Qiagen, Hilden, Germany) according to the manufacturer’s instructions.

Changes to the abundance of transcripts for key nitrogen cycling processes (archaeal and bacterial ammonia monooxygenase (*amoA*), archaeal and bacterial nitrite reductase (*nirK* and *nirS*) and hydrazine oxidoreductase (*hzo*) were analysed *via* quantitative PCR (qPCR). In addition, as proxies for bacterial and archaeal abundance and activity, archaeal and bacterial 16S rRNA genes and 16S RNA were also quantified. All qPCR data were generated using an ABI 7000 sequence detection system (Applied Biosystems) and QuantiFast SYBR Green PCR Kit (Qiagen). Reaction mixtures contained 3µL DNA or 0.75µL cDNA, 10µL Quantifast Mastermix and the primer concentrations within Table S2 in a total of 20µL. Assays contained a standard curve containing 10^2^ to 10^8^ amplicons µL^-1^ cDNA or DNA. Standard curves for each primer pair were constructed using cloned sequences. For RT qPCR, standard curves were produced from cDNA following prior in vitro transcription of cloned sequences using the Ampliscribe T7 Flash kit (Epicentre) following methodologies described by Smith et al. (2006). Nucleic acids were quantified using a NanoDrop spectrophotometer (NanoDrop Technologies, Delaware). Quantities of gene and transcripts were quantified by comparison to standard curves using the ABI Prism 7000 detection software and CT (cycles to threshold) were determined using automatic analysis settings. The no-template controls were below the threshold in all experiments. For each standard curve, the slope, y intercept, coefficient of determination (r^2^) and the efficiency of amplification are reported in Table S3.

Terminal Restriction Fragment Length Polymorphism (T-RFLP) was also used to compare the impact of sediment type, fishing intensity and organic matter addition on the composition of total and active bacterial and archaeal communities. PCR amplification of 16S rRNA (cDNA) and 16S rRNA genes (DNA) were performed using the primer pair 63F (5’-CAGGCCTAACACATGCAAGTC-3’) (Marchesi et al. 1998) and 1389R (5’-ACGGGCGGTGTGTACAAG-3’) (Osborn et al. 2000) for bacteria, and Arc109F (5’-ACKGCTCAGTAACACGT-3’) (Grosskopf et al. 1998) and Arc915R (5’-GTGCTCCCCCGCCAATTCCT-3’) (Stahl and Amann 1991) for archaea. Both forward primers were labelled with the phosphoramide fluorochrome 5-carboxy-fluorescein (6-FAM). PCR amplifications were performed in volumes of 50µL containing 10µL of 5X GoTaq® Flexi Reaction Buffer, 2.5µL of 25mM MgCl2, 5µL of dNTPs, 200µM of each primer, 0.25µL of GoTaq® G2 Hot Start Polymerase and 3µL DNA or 0.75 µL cDNA. For bacterial 16S rRNA and 16S rRNA genes, the reaction mixture was incubated at 95°C for 2 min followed by 30 cycles of 95 °C for 1 min, 57 °C for 1 min and 72°C for 1 min, followed by a final extension of 72°C for 5 min. Archaeal 16S rRNA and 16S rRNA gene PCR reactions required 35 cycles. PCR products were purified with the QIAquick® PCR Purification Kit and 5 µL subjected to restriction enzyme digestion with 20 U AluI restriction enzyme at 37˚C for 3h for the bacterial PCR products, and TaqI restriction enzyme at 65˚C for 1.5h for the archaeal PCR products, in a total of 20 µl. The digested PCR products were purified with the UltraClean®-htp 96 Well PCR Clean-Up Kit (MoBio Laboratories, Inc, Carlsbad, Califormia, USA) and fragments analysed at DNA Sequencing and Services (Medical Sciences Institute, University of Dundee, Dundee, UK) using the internal size standard ROX500. Resulting electropherograms were analysed using Peak Scanner™ Software (Applied Biosystems) and T-Align (Smith et al. 2005).

**References**

Grosskopf R, Janssen PH, Liesack W. (1998). Diversity and structure of the methanogenic community in anoxic rice paddy soil microcosms as examined by cultivation and direct 16S rRNA gene sequence retrieval. Appl Environ Microbiol 64: 960–969.

Marchesi JR, Sato T, Weightman AJ, Martin TA, Fry JC, Hiom SJ, Dymock D, Wade WG. (1998) Design and evaluation of useful bacterium-specific PCR primers that amplify genes coding for bacterial 16S rRNA. Appl Environ Microbiol 64:795–799.

Osborn AM, Moore ER, Timmis KN. (2000). An evaluation of terminal-restriction fragment length polymorphism (T-RFLP) analysis for the study of microbial community structure and dynamics. Environ Microbiol 2: 39–50.

Smith CJ, Danilowicz BS, Clear AK, Costello FJ, Wilson B, Meijer W. (2005). T-Align, a web-based tool for comparison of multiple terminal restriction fragment length polymorphism profiles. FEMS Microbiol Ecol 54: 375-380.

Smith CJ, Nedwell DB., Dong LF, Osborn AM (2006). Evaluation of quantitative polymerase chain reaction-based approaches for determining gene copy and gene transcript numbers in environmental samples. Environ Microbiol 8: 804–815.

Stahl DA, Amann R. (1991). Development and application of nucleic acid probes. In: Nucleic Acid Techniques in Bacterial Systematics (Stackebrandt E., Goodfellow M. Eds.), pp.205–248 Wiley, Chichester, England.

**Supplementary Table S2.** Details of the PCR primers, target organisms and genes, qPCR cycling conditions used and reference for each qPCR assay.

| **Target**  **Organism (and gene)** | **Name of primer** | **Sequence of primer**  **(5’-3)’** | **Primer concent-ration** | **Cycling conditions** | **Reference** |
| --- | --- | --- | --- | --- | --- |
| Bacterial ammonia monooxygenase (*amoA*) | amoA1F  amoA2R | GGGGHTTYTACTGGTGGT  CCCCTCKGSAAAGCCTTCTTC | 900 nM  900 nM | 95˚C for 5 min  Then 40 cycles of 95˚C for 15 sec and 61.5˚C for 1 min.  Followed by dissociation step. | Stephen et al., 1996  Hornek et al., 2006 |
| Archaeal ammonia monooxygenase  (*amoA*) | Arch-amoA-for  Arch-amoA-rev | CTGAYTGGGCYTGGACATC  TTCTTCTTTGTTGCCCAGTA | 300 nM  300 nM | 95˚C for 5 min  Then 40 cycles of 95˚C for 15 sec and 58.5˚C for 1 min.  Followed by dissociation step. | Wuchter et al., 2006 |
| Bacterial nitrite reductase  (*nirS*) | nirS1F  nirS3R | CCTAYTGGCCGCCRCART  GCCGCCGTCRTGVAGGAA | 900 nM  900 nM | 95˚C for 5 min  Then 40 cycles of 95˚C for 15 sec and 62˚C for 1 min.  Followed by dissociation step. | Braker et al., 1998 |
| Archaeal nitrite reductase  *(nirK*) | AnirKa-58F  AnirKb-579R | ACBYTATTCGGAAGYACATACACA  GYMATTCCGTACATKCCGGA | 400 nM  400 nM | 95˚C for 5 min  Then 40 cycles of 95˚C for 15 sec; 50˚C for 30 sec; 72˚C for 45 sec.  Followed by dissociation step. | Lund et al., 2006 |
| Bacterial hydrazine oxidoreductase  (*hzo*) | HzoF1  hzocl1R2 | GTGCATGGTCAATTGAAAG  ACTCCAGATRTGCTGACC | 300 nM  300 nM | 95˚C for 5 min  Then 40 cycles of 95˚C for 15 sec; 53˚C for 30 sec; 72˚C for 45 sec.  Followed by dissociation step. | Li et al. 2013  Schmid et al. 2008 |
| Universal Bacterial 16S rRNA | Bact 1369F  Prok 1492R | CGGTGAATACGTTCYCGG  GGWTACCTTGTTACGACTT | 900 nM  300 nM | 95˚C for 5 min  Then 40 cycles of 95˚C for 15 sec and 60˚C for 1 min.  Followed by dissociation step. | Suzuki et al. 2000 |
| Universal Archaeal 16S rRNA | Parch519f  ARC915r | CAGCCGCCGCGGTAA  GTCGCTCCCCCGCCAATTCCT | 300 nM  300 nM | 95˚C for 5 min  Then 40 cycles of 95˚C for 15 sec and 63˚C for 1 min.  Followed by dissociation step. | Øvreås et al., 1997 |

**Supplementary Table S3.** Details of qPCR and RT-qPCR statistics. For each standard curve, the slope, y intercept, the efficiency of amplification and co-efficient of determination (r^2^) were determined.

| **Gene** | **Nucleic Acid** | **Slope** | **Intercept** | **Efficiency** | **R^2^** |
| --- | --- | --- | --- | --- | --- |
| Bacterial *amoA* | RNA | -3.41 | 35.06 | 96.45 % | 0.99 |
| Archaeal *amoA* | RNA | -3.07 | 31.57 | 111.71 % | 0.99 |
| Bacterial *nirS* | RNA | -3.29 | 33.07 | 101.35 % | 0.99 |
| Archaeal *nirK* | RNA | -3.17 | 33.78 | 106.76 % | 0.99 |
| Bacterial *hzo* | RNA | -3.13 | 32.33 | 108.68 % | 0.98 |
| Bacterial 16S rRNA | DNA | -3.42 | 37.26 | 96.06 % | 0.99 |
|  | RNA | -3.12 | 34.88 | 109.18 % | 0.99 |
| Archaeal 16S rRNA | DNA | -3.48 | 36.31 | 93.80 % | 0.99 |
|  | RNA | -3.55 | 36.90 | 91.29 % | 0.99 |

**ESM3. Statistical model summaries**

Summary of the statistical analyses for the 30 models examined in (A) sand (Models S1-S15) and (B) sandy mud sediments (Models S16-S30). For each statistical model, we list the initial linear regression model, the minimal adequate model with GLS estimation and a summary of the coefficient table where appropriate. The coefficients indicate the relative performance of each treatment level (Fishing Frequency: Low and High) (Enrichment: Non-enriched and Enriched) relative to the re-levelled baseline (as indicated, initial baseline: Low or Non-enriched). Coefficients ± SE and t-values are presented alongside corresponding significance values (in parentheses). Where none of the independent variables were found significant, we present the intercept only model.

**A. Sandy sediment**

**Model 1** | **Total macro-invertebrate density (counts m^-1^)**

Initial linear regression model:

lm(density ~ Fishing Frequency × Organic enrichment)

No minimal adequate model, intercept only (L-ratio = 2.54, df = 1, p = 0.11)

**Model 2** | **Total macro-invertebrate biomass (g m^-1^)**

Initial linear regression model:

lm(biomass ~ Fishing Frequency × Organic enrichment)

No minimal adequate model, intercept only (L-ratio = 3.37, df = 1, p = 0.07)

**Model 3** | **Species richness**

Initial linear regression model:

lm(richness ~ Fishing Frequency × Organic enrichment)

No minimal adequate model, intercept only (L-ratio = 1.32, df = 1, p = 0.25)

**Model 4** | **Suspension : deposit feeder ratio**

Initial linear regression model:

lm(suspension:deposit ~ Fishing Frequency × Organic enrichment)

Minimal adequate model:

gls(suspension:deposit ~ Fishing Frequency, weights = varIdent(fishing frequency), method = "REML")

Coefficients table for fishing frequency

| Intercept ± SE  (when baseline is for Low fishing frequency) | 0.22 ± 0.06, t = 3.52, p = 0.003 |
| --- | --- |
| High fishing frequency | 1.67 ± 0.27, t = 6.23, p < 0.0001 |

**Model 5** | **Density of different sediment reworking groups**

Initial linear regression model:

lm(density ~ Fishing Frequency × Organic enrichment × Sediment reworking group)

Minimal adequate model:

gls(density ~ Fishing Frequency × Sediment reworking group, weights = varIdent(fishing frequency × Sediment reworking group), method = "REML")

**Model 6** | **Biomass of different sediment reworking groups**

Initial linear regression model:

lm(biomass ~ Fishing Frequency × Organic enrichment × Sediment reworking group)

Minimal adequate model:

gls(biomass ~ Organic enrichment + Sediment reworking group, weights = varIdent(fishing frequency × sediment reworking group), method = "REML")

Coefficients table for organic enrichment

| Intercept ± SE  (when baseline is for Non-enriched) | 0.17 ± 0.05, t = 3.52, p = 0.0007 |
| --- | --- |
| Enriched | -0.13 ± 0.07, t = -2.03, p = 0.05 |

Coefficients table for sediment reworking groups

| Intercept ± SE  (when baseline is for epifauna) | 0.17 ± 0.05, t = 3.52, p = 0.0007 |
| --- | --- |
| Surficial modifier (SM) | 0.42 ± 0.25, t = 1.70, p = 0.09 |
| Conveyor (C) | 3.68 ± 1.13, t = 3.26, p = 0.002 |
| Biodiffusor (B) | 5.45 ± 1.67, t = 3.26, p = 0.002 |
| Regenerator (R) | 3.53 ± 1.12, t = 3.15, p = 0.002 |

**Model 7** | **Abundance of bacterial denitrifier (*nirS*) (copies g^-1^ sediment)**

Initial linear regression model:

lm(nirS ~ Fishing Frequency × Organic enrichment)

Minimal adequate model:

gls(nirS ~ Fishing Frequency + Organic enrichment, weights = varIdent(fishing frequency), method = "REML")

Coefficients table for organic enrichment

| Intercept ± SE  (when baseline is for Non-enriched) | 647.34 ± 151.01, t = 4.29, p = 0.005 |
| --- | --- |
| Enriched | 2084.21 ± 261.45, t = 7.97, p = 0.0002 |

Coefficients table for fishing frequency

| Intercept ± SE  (when baseline is for Low) | 647.34 ± 151.01, t = 4.29, p = 0.005 |
| --- | --- |
| High | 4991.80 ± 2448.42, t = 2.04, p = 0.09 |

**Model 8** | **Abundance of archaeal denitrifier (A*nirKa*) (copies g^-1^ sediment)**

Initial linear regression model:

lm(AnirKa ~ Fishing Frequency × Organic enrichment)

Minimal adequate model:

glm(AnirKa ~ Fishing Frequency), weights = varIdent(fishing frequency × Organic enrichment), method = "REML"

Coefficients table for fishing frequency

| Intercept ± SE  (when baseline is for Low fishing frequency) | 6414.91 ± 1762.30, t = 3.64, p = 0.004 |
| --- | --- |
| High fishing frequency | 40092.02 ± 13388.06, t = 2.99, p = 0.01 |

**Model 9** | **Abundance of archaeal anammox (*hzo*) (copies g^-1^ sediment)**

Initial linear regression model:

lm(hzo ~ Fishing Frequency × Organic enrichment)

Minimal adequate model:

glm(hzo ~ Fishing Frequency), weights = varIdent(fishing frequency, method = "REML")

Coefficients table for fishing frequency

| Intercept ± SE  (when baseline is for Low fishing frequency) | 390.24 ± 172.94, t = 2.26, p = 0.05 |
| --- | --- |
| High fishing frequency | 1517.26 ± 500.05, t = 3.03, p = 0.01 |

**Model 10** | **Abundance of archaeal nitrifier (AOA *amoA*) (copies g^-1^ sediment)**

Initial linear regression model:

lm(AOA amoA ~ Fishing Frequency × Organic enrichment)

No minimal adequate model, intercept only ( L-ratio = 1.25, df = 1, p = 0.26)

**Model 11** | **Abundance of bacterial nitrifier (AOB *amoA*) (copies g^-1^ sediment)**

Initial linear regression model:

lm(AOB amoA ~ Fishing Frequency × Organic enrichment)

No minimal adequate model, intercept only (L-ratio = 2.50, df = 1, p = 0.11)

**Model 12** | **AOB:AOA *amoA* ratio**

Initial linear regression model:

lm(AOB : AOA ~ Fishing Frequency × Organic enrichment)

No minimal adequate model, intercept only (L-ratio = 3.28, df = 1, p = 0.07)

**Model 13** | **Nitrite ([NO_2_-N], μM**

Initial linear regression model:

lm([NO_2_-N] ~ Fishing Frequency × Organic enrichment)

Minimal adequate model:

gls([NO_2_-N] ~ Fishing Frequency, weights = varIdent(fishing frequency), method = "REML")

Coefficients table for fishing frequency

| Intercept ± SE  (when baseline is for Low fishing frequency) | 0.31 ± 0.07, t = 4.52, p = 0.0003 |
| --- | --- |
| High fishing frequency | 17.76 ± 6.53, t = 2.72, p = 0.01 |

**Model 14** | **Nitrate ([NO_3_-N], μM**

Initial linear regression model:

lm([NO_3_-N] ~ Fishing Frequency × Organic enrichment)

Minimal adequate model:

glm([NO_3_-N] ~ Fishing Frequency), weights = varIdent(fishing frequency× Organic enrichment), method = "REML")

Coefficients table for fishing frequency

| Intercept ± SE  (when baseline is for Low fishing frequency) | 10.25 ± 1.97, t = 5.21, p < 0.0001 |
| --- | --- |
| High fishing frequency | 20.95 ± 3.13, t = 6.69, p < 0.0001 |

**Model 15** | **Ammonium ([NH_4_-N], μM)**

Initial linear regression model:

lm([NH_4_-N]~ Fishing Frequency × Organic enrichment)

No minimal adequate model, intercept only ( L-ratio = 1.12, df = 1, p = 0.29)

**B. sandy Mud sediment**

**Model 16** | **Total macro-invertebrate density (counts m^-1^)**

Initial linear regression model:

lm(density ~ Fishing Frequency × Organic enrichment)

Minimal adequate model:

gls(density ~ Fishing Frequency + Organic enrichment, weights = varIdent(fishing frequency), method = "REML")

Coefficients table for organic enrichment

| Intercept ± SE  (when baseline is for Non-enriched) | 3.68 ± 0.86, t = 4.28, p = 0.0005 |
| --- | --- |
| Enriched | 2.44 ± 1.17, t = 2.09, p = 0.05 |

Coefficients table for fishing frequency

| Intercept ± SE  (when baseline is for Low fishing frequency) | 3.68 ± 0.86, t = 4.28, p = 0.0005 |
| --- | --- |
| High fishing frequency | 4.20 ± 1.74, t = 2.41, p = 0.03 |

**Model 17** | **Species richness**

Initial linear regression model:

lm(richness ~ Fishing Frequency × Organic enrichment)

Minimal adequate model:

gls(richness ~ Fishing Frequency + Organic enrichment, weights = varIdent(fishing frequency), method = "REML")

Coefficients table for organic enrichment

| Intercept ± SE  (when baseline is for Non-enriched) | 2.25 ± 0.40, t = 5.61, p < 0.0001 |
| --- | --- |
| Enriched | 1.71 ± 0.55, t = 3.09, p = 0.01 |

Coefficients table for fishing frequency

| Intercept ± SE  (when baseline is for Low fishing frequency) | 2.25 ± 0.40, t = 5.61, p < 0.0001 |
| --- | --- |
| High fishing frequency | 1.90 ± 0.95, t = 2.01, p = 0.06 |

**Model 18** | **Total macro-invertebrate biomass (g m^-1^)**

Initial linear regression model:

lm(biomass~ Fishing Frequency × Organic enrichment)

No minimal adequate model, intercept only (L-ratio = 1.29, df = 1, p = 0.26)

**Model 19** | **Suspension : deposit feeder ratio**

Initial linear regression model:

lm(suspension:deposit ~ Fishing Frequency × Organic enrichment)

Minimal adequate model:

No minimal adequate model, intercept only (L-ratio = 12.33, df = 1, p = 0.77)

**Model 20** | **Density of different sediment reworking groups**

Initial linear regression model:

lm(density ~ Fishing Frequency × Organic enrichment × Sediment reworking group)

Minimal adequate model:

gls(density ~ Fishing Frequency + Organic enrichment + Sediment reworking group, weights = varIdent(fishing frequency × organic enrichment), method = "REML")

Coefficients table for organic enrichment

| Intercept ± SE  (when baseline is for Non-enriched) | 14.64 ± 5.74, t = 2.55, p = 0.01 |
| --- | --- |
| Enriched | 19.30 ± 7.94, t = 2.43, p = 0.02 |

Coefficients table for fishing frequency

| Intercept ± SE  (when baseline is for Low fishing frequency) | 14.64 ± 5.74, t = 2.55, p = 0.01 |
| --- | --- |
| High fishing frequency | 31.77 ± 10.40, t = 3.05, p = 0.004 |

Coefficients table for sediment reworking groups

| Intercept ± SE  (when baseline is for Surficial modifier, SM) | 14.64 ± 5.74, t = 2.55, p = 0.01 |
| --- | --- |
| Conveyor, C | -15.99 ± 7.70, t = -2.07, p = 0.04 |
| Biodiffusor, B | 58.33 ± 7.70, t = 7.57, p < 0.0001 |

**Model 21** | **Biomass of different sediment reworking groups**

Initial linear regression model:

lm(biomass ~ Fishing Frequency × Organic enrichment × Sediment reworking group)

Minimal adequate model:

gls(biomass ~ Sediment reworking group, weights = varIdent(fishing frequency × sediment reworking group), method = "REML")

Coefficients table for sediment reworking groups

| Intercept ± SE  (when baseline is for Surficial modifier, SM) | 1.24 ± 0.53, t = 2.35, p = 0.02 |
| --- | --- |
| Conveyor, C | -1.19 ± 0.53, t = -2.24, p = 0.03 |
| Biodiffusor, B | 8.76 ± 5.22, t = 1.68, p = 0.1 |

**Model 22** | **Abundance of bacterial denitrifier (*nirS*) (copies g^-1^ sediment)**

Initial linear regression model:

lm(nirS ~ Fishing Frequency × Organic enrichment)

Minimal adequate model:

No minimal adequate model, intercept only (F = 0.005, df = 17, p = 0.94)

**Model 23** | **Abundance of archaeal denitrifier (A*nirKa*) (copies g^-1^ sediment)**

Initial linear regression model:

lm(AnirKa ~ Fishing Frequency × Organic enrichment)

Minimal adequate model:

lm(AnirKa ~ Fishing Frequency + Organic enrichment, method = "REML")

Coefficients table for organic enrichment

| Intercept ± SE  (when baseline is for Non-enriched) | 686214 ± 195412, t = 3.51, p = 0.003 |
| --- | --- |
| Enriched | -434505 ± 218140, t = -1.99, p = 0.06 |

Coefficients table for fishing frequency

| Intercept ± SE  (when baseline is for Low fishing frequency) | 686214 ± 195412, t = 3.51, p = 0.003 |
| --- | --- |
| High fishing frequency | 424822 ± 216790, t = -1.99, p = 0.07 |

**Model 24** | **Abundance of archaeal anammox (*hzo*) (copies g^-1^ sediment)**

Initial linear regression model:

lm(hzo ~ Fishing Frequency × Organic enrichment)

Minimal adequate model:

No minimal adequate model, intercept only (F = 0.33, df = 17, p = 0.57)

**Model 25** | **Abundance of archaeal nitrifier (AOA *amoA*) (copies g^-1^ sediment)**

Initial linear regression model:

lm(AOA amoA ~ Fishing Frequency × Organic enrichment)

No minimal adequate model, intercept only (F = 0.46, df = 17, p = 0.51)

**Model 26** | **Abundance of bacterial nitrifier (AOB *amoA*) (copies g^-1^ sediment)**

Initial linear regression model:

lm(AOB amoA ~ Fishing Frequency × Organic enrichment)

No minimal adequate model, intercept only (F = 0.17, df = 17, p = 0.68)

**Model 27** | **AOB:AOA *amoA* ratio**

Initial linear regression model:

lm(AOB:AOA ~ Fishing Frequency × Organic enrichment)

Minimal adequate model:

lm(AOB:AOA ~ Organic enrichment, method = "REML")

Coefficients table for organic enrichment

| Intercept ± SE  (when baseline is for Non-enriched) | 2.05 ± 0.41, t = 5.02, p = 0.0001 |
| --- | --- |
| Enriched | 1.99 ± 0.55, t = 3.65, p = 0.002 |

**Model 28** | **Nitrite ([NO_2_-N], μM**

Initial linear regression model:

lm([NO_2_-N] ~ Fishing Frequency × Organic enrichment)

No minimal adequate model, intercept only (L-ratio = 1.37, df = 1, p = 0.24)

**Model 29** | **Nitrate ([NO_3_-N], μM**

Initial linear regression model:

lm([NO_3_-N] ~ Fishing Frequency × Organic enrichment)

No minimal adequate model, intercept only (F = 0.49, df = 19, p = 0.49)

**Model 30** | **Ammonium ([NH_4_-N], μM)**

Initial linear regression model:

lm([NH_4_-N]~ Fishing Frequency × Organic enrichment)

No minimal adequate model, intercept only (L-ratio = 1.73, df = 1, p = 0.19)

**ESM4. Analysis of microbial community data in PERMANOVA package**

**Supplementary FigureS2.** Non-metric multidimensional scaling (nMDS) ordination of Bray-Curtis resemblance matrix calculated from T-RFLPs for (a) archaeal 16S rRNA gene abundance and (b) bacterial 16S rRNA gene abundance in **sand**. Contrasting levels of bottom fishing frequency (open symbol low, closed symbol high) and organic matter enrichment (circle non-enriched, square enriched) are presented.

(a) Archaeal 16S rRNA gene abundance in sand

**
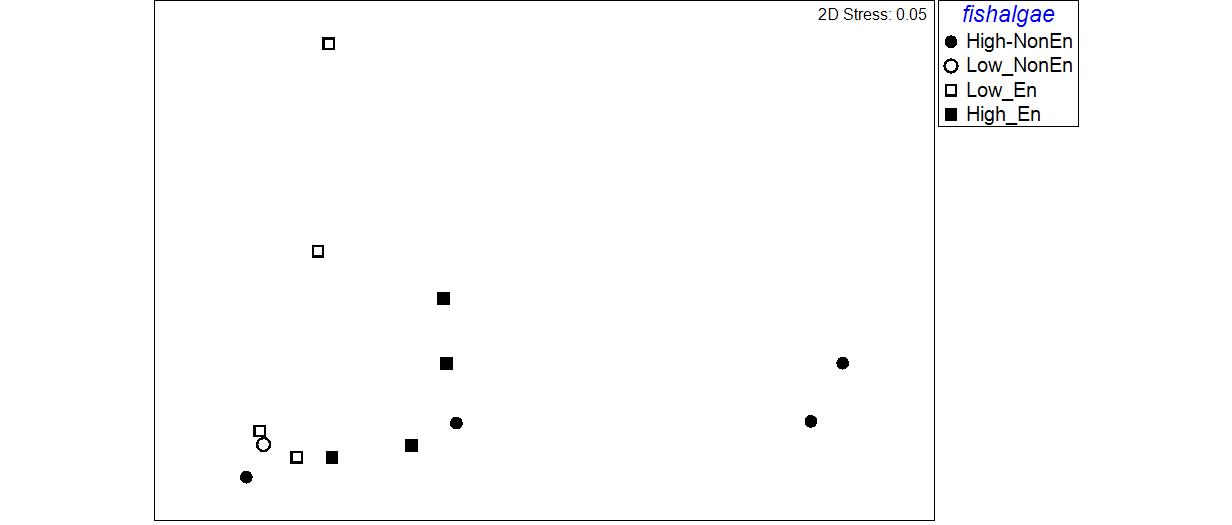
**

(b) Bacterial 16S rRNA gene abundance in sand

**
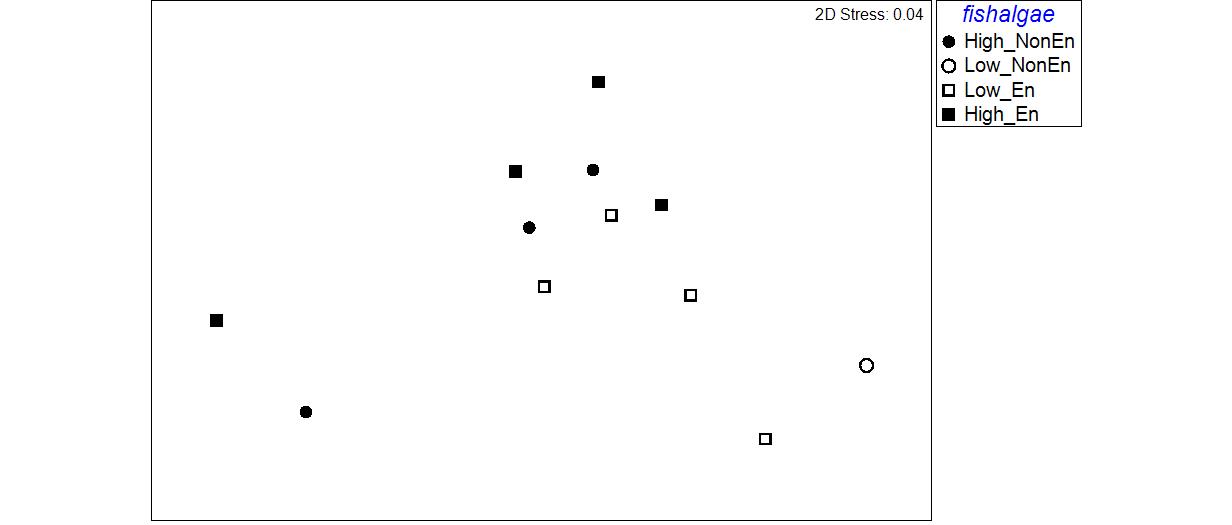
**

**Supplementary FigureS3.** Non-metric multidimensional scaling (nMDS) ordination of Bray-Curtis resemblance matrix calculated from T-RFLPs for (a) archaeal 16S rRNA gene abundance and (b) bacterial 16S rRNA gene abundance in **sandy mud**. Contrasting levels of bottom fishing frequency (open symbol low, closed symbol high) and organic matter enrichment (circle non-enriched, square enriched) are presented.

(a) Archaeal 16S rRNA gene abundance in sandy Mud

**
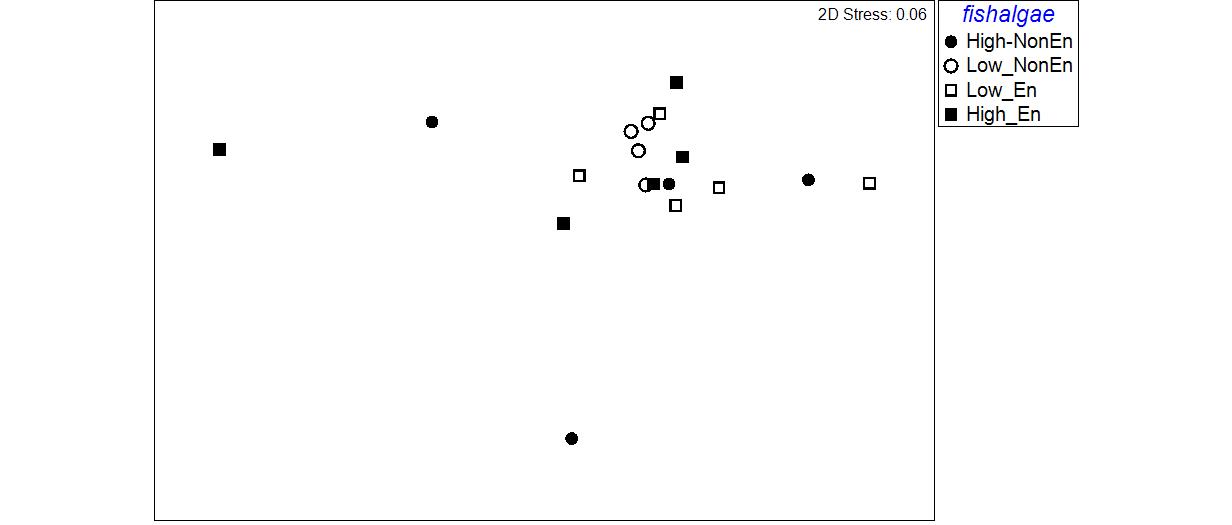
**

(b) Bacterial 16S rRNA gene abundance in sandy Mud

**
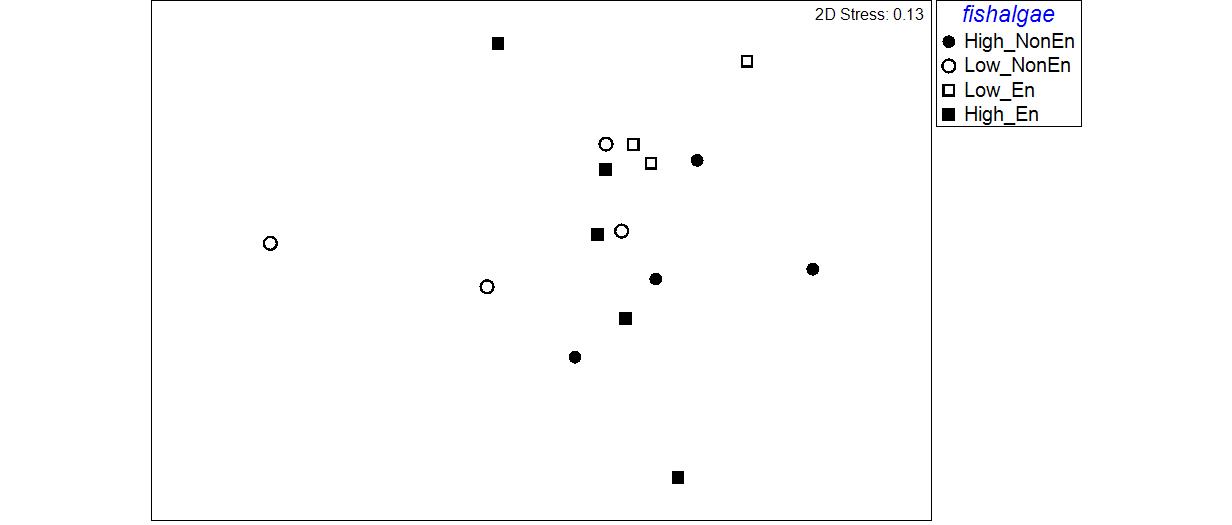
**

**ESM5. Analysis of macro-invertebrate community data in PERMANOVA package**

**Table S3.** The similarity percentage (SIMPER) dissimilarity tables (up to 90% of cumulative differences) of taxa (I) density and (II) biomass in **sandy mud sites** that experienced contrasting levels of fishing frequency (Levels: low and high fishing frequency). Information on species feeding mode/s (SDF for surface deposit feeder; SubDF for subsurface deposit feeder; ASF for active suspension feeder; PSF for passive suspension feeder; Pred for predator; Scav for scavenger; Det for detritivore; feeding mode was not allocated to taxon level higher than genus and are denoted by “-”), sediment reworking functional type (E for epifauna; SM for surficial modifiers; UC/DC for upward and downward conveyors; B for biodiffusors; and R for regenerators) and mobility (1 for organisms that live in fixed tubes; 2 indicates limited movement; 3 indicates slow, free movement through the sediment matrix; 4 indicates free movement via burrow system) are provided.

| **I. Groups tested: Taxon density between low and high fishing disturbance in sandy mud sediment** | | | | | | |
| --- | --- | --- | --- | --- | --- | --- |
| **Species** | **Feeding mode** | **^3^Sediment reworking functional type** | **^3^Mobility** | **Low disturbance** | **High disturbance** | **Contr. Diss. (%)** |
| *Nephtys incisa* | ^1^SDF, SubDF | B | 3 | 0.98 | 1.08 | 13.39 |
| *Nephtys* sp. | ^1^Pred, Scav | B | 3 | 0.27 | 0.48 | 9.67 |
| *Goneplax rhomboides* | ^2^SDF, SubDF | B | 4 | 0.54 | 0.10 | 7.84 |
| *Lagis koreni* | ^1^SubDF | UC/DC | 1 | 0.17 | 0.69 | 7.69 |
| *Lumbrineris* sp. | ^1^Pred, Scav | B | 3 | 0.30 | 0.38 | 6.93 |
| *Phoronis sp.* | ^1^PSF, ASF | SM | 1 | 0.24 | 0.34 | 5.40 |
| *Magelona sp.* | ^2^SDF | SM | 2 | 0.34 | 0.00 | 4.99 |
| *Sthenelais limicola* | ^2^Pred, Scav | B | 3 | 0.00 | 0.34 | 4.38 |
| *Ophelina acuminata* | ^2^SubDF | B | 3 | 0.24 | 0.14 | 4.33 |
| *Abra alba* | ^1^PSF, ASF, SDF, SubDF | SM | 2 | 0.00 | 0.30 | 4.22 |
| *Scalibregma inflatum* | ^1^SDF, SubDF | B | 4 | 0.00 | 0.44 | 4.19 |
| *Nucula hanleyi* | ^1^SubDF, Det | SM | 3 | 0.20 | 0.10 | 3.13 |
| *Magelona johnstoni* | ^2^SDF | SM | 2 | 0.00 | 0.27 | 2.59 |
| *Chaetozone* sp. | ^2^SDF | SM | 2 | 0.10 | 0.10 | 2.19 |
| *Abra prismatica* | ^1^PSF, ASF, SDF, SubDF | SM | 2 | 0.00 | 0.24 | 2.18 |
| *Glycera oxycephala* | ^1^Pred, Scav | B | 3 | 0.00 | 0.20 | 1.84 |
| *Corbula gibba* | ^1^PSF, ASF | SM | 2 | 0.00 | 0.10 | 1.68 |
| *Spisula* sp*.* | ^1^PSF, ASF | SM | 2 | 0.00 | 0.10 | 1.59 |
| *Glycera alba* | ^1^Pred, Scav | B | 3 | 0.10 | 0.00 | 1.23 |
| Spionidae | - | UC/DC | 2 | 0.00 | 0.10 | 1.16 |
| **II. Groups tested: Taxon biomass between low and high fishing disturbance in sandy Mud sediment** | | | | | | |
| **Species** | **Feeding mode** | **^4^Sediment reworking functional type** | **^4^Mobility** | **Low disturbance** | **High disturbance** | **Contr. Diss. (%)** |
| *Goneplax rhomboides* | ^2^SDF, SubDF | B | 4 | 0.42 | 0.16 | 32.76 |
| *Nepthys sp.* | ^1^Pred, Scav | B | 3 | 0.19 | 0.20 | 13.63 |
| *Abra alba* | ^1^PSF, ASF, SDF, SubDF | SM | 2 | 0.00 | 0.07 | 7.55 |
| *Nucula hanleyi* | ^1^SubDF, Det | SM | 3 | 0.11 | 0.01 | 6.33 |
| *Abra prismatica* | ^1^PSF, ASF, SDF, SubDF | SM | 2 | 0.00 | 0.05 | 4.32 |
| *Lumbrineris sp.* | ^1^Pred, Scav | B | 3 | 0.02 | 0.02 | 4.07 |
| *Lagis koreni* | ^1^SubDF | UC/DC | 1 | 0.01 | 0.03 | 3.89 |
| *Glycera alba* | ^1^Pred, Scav | B | 3 | 0.03 | 0.00 | 2.68 |
| *Glycera unicornis* | ^1^Pred, Scav | B | 3 | 0.08 | 0.00 | 2.56 |
| *Corbula gibba* | ^1^PSF, ASF | SM | 2 | 0.00 | 0.02 | 1.79 |
| *Abyssoninoe hibernica* | ^2^Pred, Scav | B | 3 | 0.00 | 0.01 | 1.63 |
| *Scalibregma inflatum* | ^1^SDF, SubDF | B | 4 | 0.000 | 0.01 | 1.63 |
| *Cerebratulus* sp. | ^2^Pred, Scav | B | 3 | 0.20 | 0.00 | 0.52 |

**Data sources**

**^1.^** MarLIN, 2006. BIOTIC - Biological Traits Information Catalogue. Marine Life Information Network. Plymouth: Marine Biological Association of the United Kingdom. [03/07/2017] Available from <www.marlin.ac.uk/biotic>

^2.^ Biological traits database developed under the BENTHIS (Benthic Ecosystem Fisheries Impact Studies) project. [16/07/2016]. http://www.benthis.eu/en/benthis/Results.htm

^3.^ Queiros AM, Birchenough SNR, Bremner J, et al (2013) A bioturbation classification of European marine infaunal invertebrates. Ecol Evol 3: 3958-3985
